# Groundwater quality assessment for agricultural utilizing indexical and machine learning techniques in Ouled Djellal Aquifer, Southern Algeria

**DOI:** 10.1038/s41598-026-38208-0

**Published:** 2026-02-10

**Authors:** Ali Athamena, Aissam Gaagai, Hani Amir Aouissi, Hamza Cheniti, Halima Belalite, Billel Touati, Habibi Yahyaoui, Feriel Kheira Kebaili, Sabrina Ziad, Salah Elsayed, Ahmed Elbeltagi, Ali Salem, Zaher Mundher Yaseen, Mohamed Gad

**Affiliations:** 1 Department of Geology, Institute of Earth and Universe Sciences, University of Batna 2, Fesdis, 05078 Algeria; 2 Scientific and Technical Research Center on Arid Regions (CRSTRA), Biskra, 07000 Algeria; 3 Environmental Research Center (CRE), Annaba, 23000 Algeria; 4 Department of Mining Engineering, Metallurgy and Materials, National Higher School of Technology and Engineering, Annaba, 23005 Algeria; 5 Department of Earth and Universe Sciences, Institute of Natural and Life Science, Mila University Center, Mila, 43000 Algeria; 6 School of Earth and Space Sciences, University of Science and Technology of China, Hefei, 230026 China; 7 Laboratory of Natural Hazards and Territorial Planning LRNAT, Institute of Earth and Universe Sciences, University of Batna 2, Batna, 05000 Algeria; 8 Department of Industrial Chemistry, University of Biskra, P.O. Box 145, Biskra, 07000 Algeria; 9 Agricultural Engineering, Evaluation of Natural Resources Department, Environmental Studies and Research Institute, University of Sadat City, Sadat City, 32897 Minufiya Egypt; 10 Agricultural Engineering Department, Faculty of Agriculture, Mansoura University, Mansoura, 35516 Egypt; 11 Civil Engineering Department, Faculty of Engineering, Minia University, Minya, 61111, Egypt; 12 Structural Diagnostics and Analysis Research Group, Faculty of Engineering and Information Technology, University of Pecs, Pécs, Hungary; 13 Civil and Environmental Engineering Department, King Fahd University of Petroleum & Minerals, Dhahran, 31261 Saudi Arabia; 14 Hydrogeology, Evaluation of Natural Resources Department, Environmental Studies and Research Institute, University of Sadat City, Sadat City, 32897 Minufiya Egypt

**Keywords:** Groundwater aquifer, Irrigation water supply, Water quality assessment, Algeria, Machine learning, Environmental sciences, Hydrology

## Abstract

Groundwater represents the main water resource for irrigation in the Ouled Djellal region (southeast of Algeria). Despite the importance of groundwater in this area, its quality and sustainability remain insufficiently studied. Therefore, this study aimed to introduce an integrated analytical framework by combining multivariate statistical techniques i.e., Principal Component Analysis (PCA) and Hierarchical Ascending Classification (HAC), irrigation indices (IWQI, SAR, Na%, SSP, PS, and RSC), and machine learning (ML) models such as Artificial Neural Network (ANN), Support Vector Machine (SVM), and Multiple Linear Regression (MLR) to assess and predict groundwater quality for irrigation. The main difference with previous studies is the fact that this work applied Empirical Bayesian Kriging Regression Prediction (EBKRP) to spatialize irrigation indices derived from ML with higher precision. The approach enables cross-validation of model performance and captures complex nonlinear interactions among hydrochemical parameters. The attained results revealed that groundwater quality was varied from moderate to poor for irrigation, driven mainly by salinity and sodicity effects. In addition, the ANN model achieved the highest predictive accuracy (R² = 0.97, RMSE = 1.50), confirming its superiority in modelling complex hydrochemical behavior. The proposed modelling framework represents a methodological advancement for data-scarce arid regions, serving as a practical tool adaptable to groundwater monitoring and irrigation planning in similar regions.

## Introduction

In countries with limited surface water resources, the richness of groundwater becomes determinant. It constitutes a vital resource for domestic supply, agriculture, and other human activities, particularly in arid and semi-arid regions^1,2^. As a result, access to higher-quality water remains a fundamental priority^3,4^. Consequently, understanding the geochemistry of groundwater is essential for elucidating the mechanisms underlying water chemistry acquisition, in order to counteract any actions that may qualitatively degrade this valuable resource^5,6^. Nevertheless, in arid (and semi-arid) zones, water supply depends largely on groundwater aquifers, which are often threatened by the deterioration of their quality because of both anthropogenic and natural factors^7,8^. Thus, assessing the geochemical profile of groundwater resources is a necessary step towards their responsible and sustainable management^9,10^. The chemical composition of water reflects a multitude of complex interactions, including water-rock interactions within the geological matrix, residence time, and flow dynamics^11–13^. These processes represent key mechanisms in the acquisition of groundwater chemistry, and a better knowledge of them will facilitate more efficient exploitation and adequate protection of the resource^14,15^.

The development of multivariate statistical methods provides powerful tools for identifying the principal factors governing groundwater chemistry and contamination, thereby offering valuable support for the water resources sustainable management^16,17^. In this work, hierarchical cluster analysis (HCA) and principal component analysis (PCA) are applied to groundwater quality from the Ouled Djellal region in southeastern Algeria, to assess water quality and delineate areas potentially exposed to pollution. The resulting findings, using Geographic Information Systems (GIS), enable the mapping of contamination extent and a better understanding of the spatial distribution of key water quality parameters^18,19^. Besides, water quality index (WQI) has also emerged as a widely used indicator, distilling complex hydrochemical data into a single, synthetic score that simplifies interpretation for the public and decision-makers. This index is particularly useful tool to assess the suitability of groundwater for irrigation purposes^20^. Consequently, machine learning (ML) is becoming increasingly important in the assessment and prediction of water quality (WQ) by offering faster, more accurate, economical, and advantageous predictions compared to traditional methods, even though the latter often rely on complex, incomplete, and sometimes limited data to a few parameters^21,22^. This is specifically beneficial in situations where analytical resources are generally limited. For example, recent research showed that the use of ML models such as artificial neural networks (ANN) and multivariate regression (MR) allows for an effective estimation of the WQ^23,24^. Because ML algorithms handle large, diverse datasets (including sensor measurements and satellite imagery), which enable rapid detection of water-quality changes. This facilitates quick identification of pollution sources (industrial or agricultural)^25^, and supports more proactive management of water resource^26^. Techniques like ANN, MLR, SVM, and others, have recently demonstrated their high performance in predicting WQIs, achieving determination coefficients exceeding 0.97 and extremely high classification accuracy^27^. The application of ML also allows for the implementation of real-time alert and continuous evaluation systems for WQ, which facilitates water resources integrated management^28^. Recent publications in the field of ML, indicated that the use of this technology has now become essential for modernizing water quality forecasting^29^.

In recent years, the Ouled Djellal region has experienced increasing water scarcity^30^. Boreholes are running dry, and irrigated water is lacking, and this situation threatens local agriculture particularly date palm cultivation making groundwater reserves a vital pillar for local communities, livestock, and vegetation. Nevertheless, the current state of groundwater, both in terms of quality and quantity, jeopardizes the sustainability of these populations and their primary sources of livelihood. Table 1 summarized an important recent works on water quality assessment and irrigation suitability which highlighted their methodology (model used), main findings, and limitations.

**Table 1:** Summary of recent works on water quality and irrigation suitability (methodology, findings, and limitations).

Study	List of Models Used	Main Findings	Limitations (Research Gaps)	Country (Study Area)
M’nassri et al., 2022^31^	IWQI, ANN, SVM	ML models improved IWQI prediction accuracy (R² > 0.9).	Focused on single index (IWQI) without multivariate integration.	Tunisia (central)
Jafar et al., 2023^32^	MLR, ML (SVM, RF)	ML models outperformed regression in predicting drinking WQI.	Limited dataset; no irrigation-focused indices.	Iraq (Al-Seine Lake)
Gaagai et al., 2023^33^	PCA, ANN, GIS	Combined hydrochemistry and ML for groundwater quality mapping.	Did not evaluate multiple IWQIs or model generalization.	Algeria (Doucen Plain)
Trabelsi & Bel Hadj Ali, 2022^29^	ANN, IWQI	Demonstrated ANN superiority for irrigation water quality.	No integration with geostatistics or multi-index validation.	Tunisia (Medjerda Basin)
Palabıyık & Akkan, 2024^24^	MLR, MLP, PCA	MLP achieved best WQI prediction (R² = 0.97).	Focused on single index (WQI); lacked spatial prediction.	Turkey (Aksu Creek)
**Our Study**	PCA, HAC, IWQIs, ANN, SVM, MLR, EBKRP	Integrated statistical-ML-geostatistical approaches; achieved R² = 0.97 for IWQI.	Introduces cross-model validation and high-resolution spatial mapping, which was the first application in the region.	Algeria (Ouled Djellal)

In order to evaluate irrigation water suitability in the Ouled Djellal region, this study introduce the integration between water quality indices (WQIs) and GIS-based machine learning approaches. Therefore, this study was conducted to (i) evaluate the influence of natural processes and anthropogenic pressures on groundwater quality in Ouled Djellal using physicochemical parameters and groundwater facies, Chloro-alkaline indices (CAI 1 and CAI 2), multiple graphical and statistical approaches, (ii) assess irrigation water quality using IWQIs, and (iii) develop and test machine learning models (ANN, SVM, MLR) integrated with GIS to predict and interpret irrigation suitability. The results of this study can guide local water management authorities in adopting sustainable irrigation practices, while reducing potential risks to human health and the surrounding ecosystems.

## Materials & methods

### Research area

**Fig. 1:**
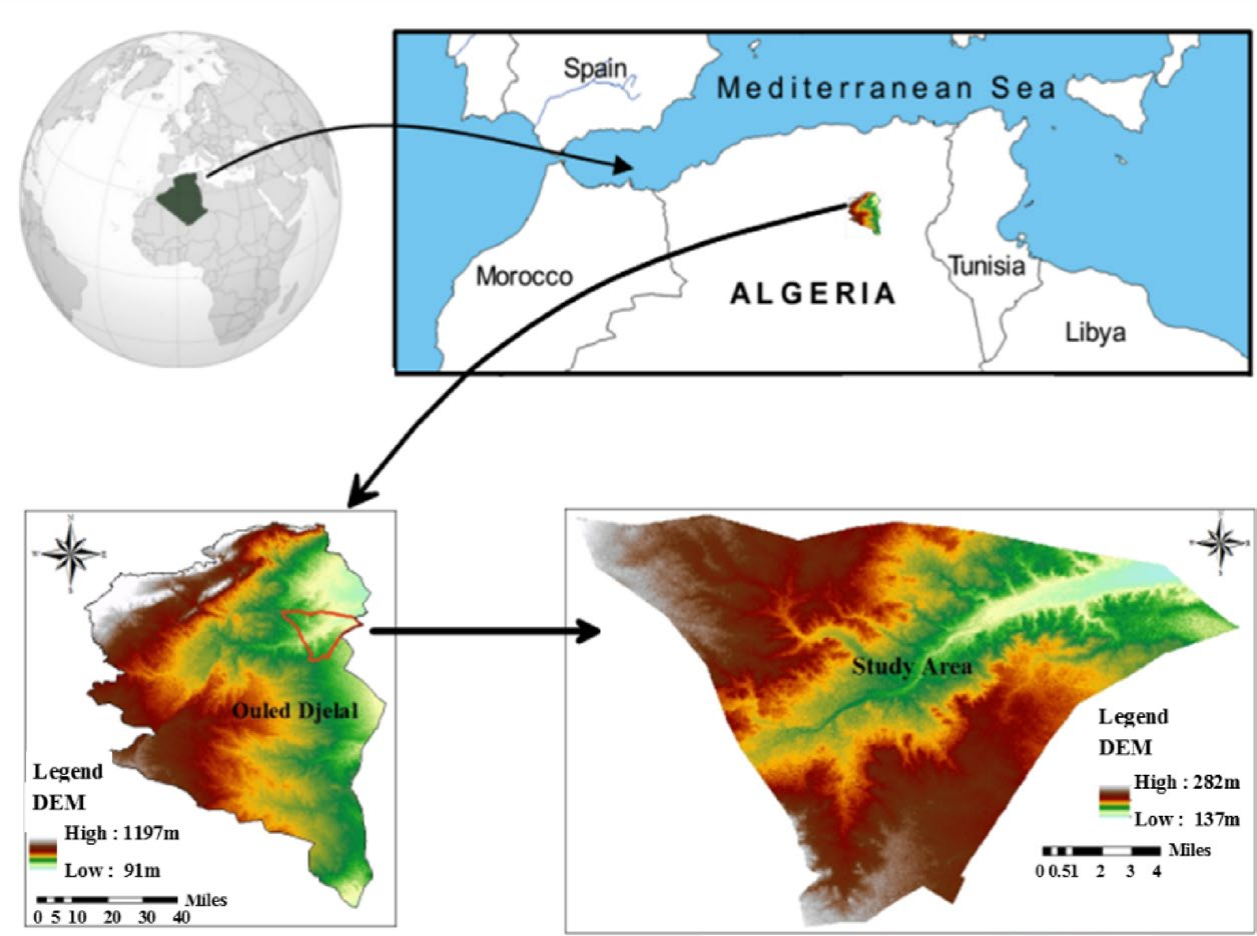
Map of the study area (Ouled Djelall, North Sahara Algeria). Map created using ArcGIS Pro 2.8.8 (Esri; https://www.esri.com/arcgis/about-arcgis).

The town of Ouled Djellal is located in the southeastern part of Algeria, bounded to northeast by the wilaya of Biskra. It covers an area of ​​ about 326.6 km^2^. It is border by the municipality of Doucen and the municipality of Chaiba in the north and northwest and by the commune of besbes and the commune of Sidi Khaled in the south and south-west. While it is border by the wilaya of El-Oued in the east (Fig. 1). From a topographic point of view, Ouled Djellal is part of the pre-Saharan region. Its relief is not very rugged and is characterized by the dominance of large areas, the average altitude is about 200 m. Hydrographically, the town is drained to the south by an important temporary wadi (Wadi Djedi). The wadi’s flow is irregular and practically zero. Other less important wadis also pass through the area of Oued Besbes, Oued Diel and Oued Rtem. Regarding the climate, the Ouled-Djellal region belongs to the Mediterranean climate type with a Saharan bioclimatic influence. It is characterized by hot and dry summers (temperature between 35 °C and 45 °C during the day, and between 25 °C and 35 °C at night), and cold, dry winters (temperature between 10 and 20 °C during the day, and between − 2 and 5 °C at night). Rainfall is very low and irregularly distributed in time and space, with the average rainfall (annual) approximately 126.2 mm, which reflecting arid to semi-arid conditions.

### Geological and hydrogeological setting

**Fig. 2:**
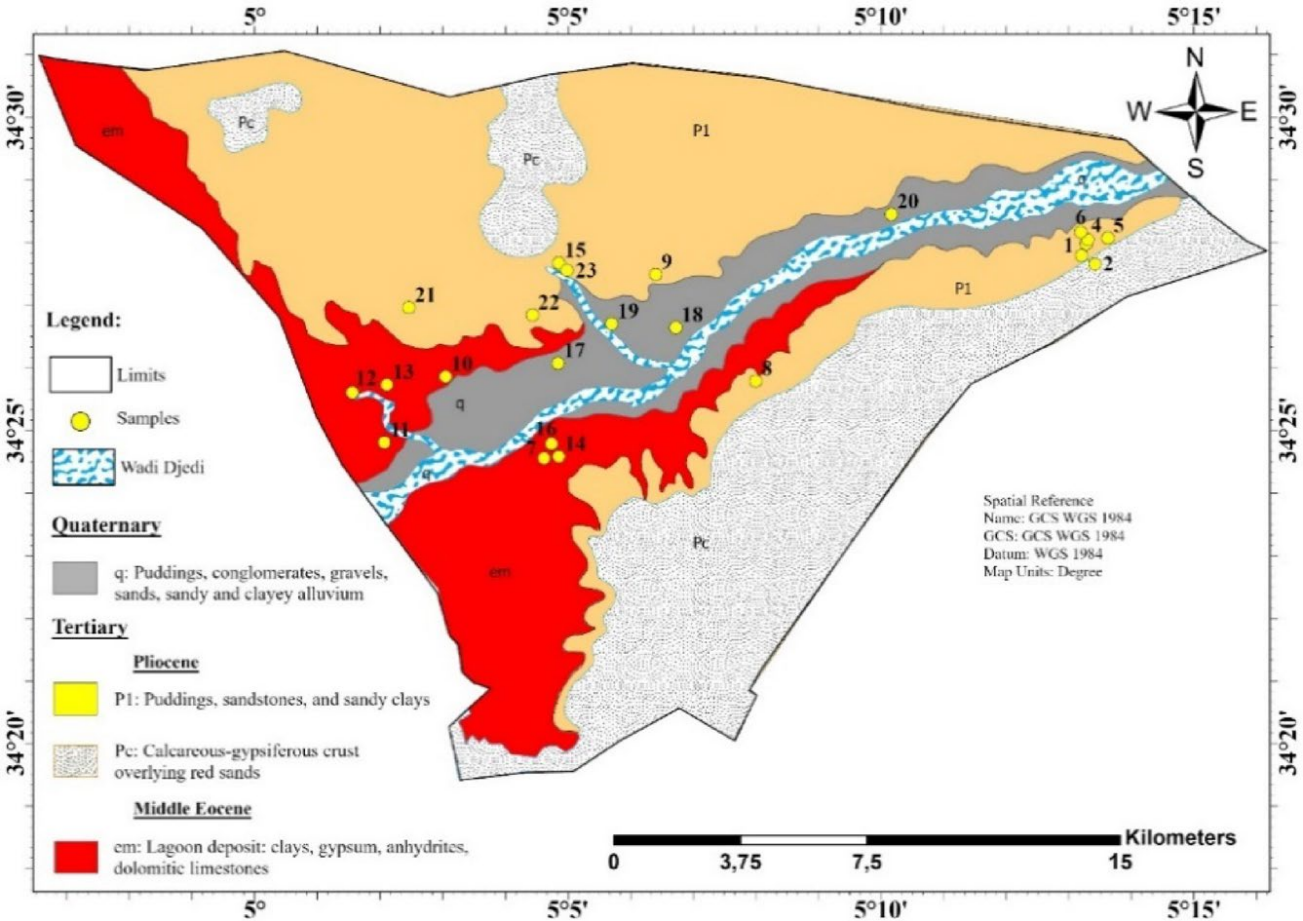
Geological map of Biskra containing our water samples. Map created using ArcGIS Pro 2.8.8 (Esri; https://www.esri.com/arcgis/about-arcgis).

**Fig. 3:**
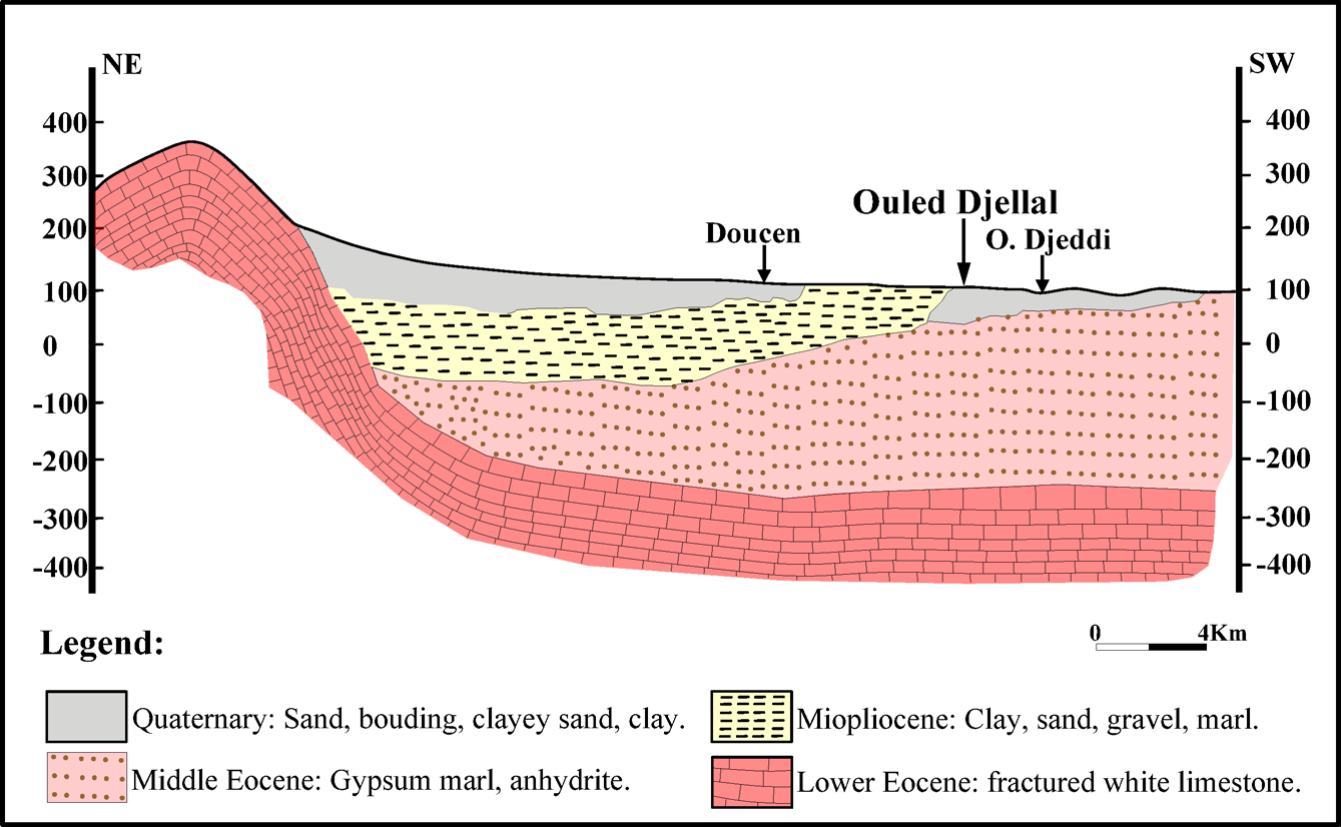
Schematic geological cross-section of the study area (made by the authors). Map created using ArcGIS Pro 2.8.8 (Esri; https://www.esri.com/arcgis/about-arcgis).

Geological and hydrogeological studied have made^34^ to highlight the different types of geological formations. Several aquifer reservoirs exist, with a significant variation in importance because of the differences in their geological structure, lithological composition, and the ease of their exploitation (Fig. 2). The Ouled Djellal region belongs to the vast sedimentary basin of the Lower Sahara, specifically in the Zab plain, which located at the junction between the southernmost tip of the Saharian Atlas, the northern Saharian basin, and the southwestern edge of the Aurès. Structurally, the region juxtaposes two sectors as the following: (i) from the north, a folded zone of the Saharian Atlas with SW-NE oriented structures inherited from the Alpine orogeny; (ii) from the south, the Saharian collapse zone characterized by depressions filled with recent formations. The transition between these domains is marked by a large tectonic structure activated after the uplift of the Atlas, called the Saharian flexure or southern Atlas flexure. Ouled Djellal is predominantly composed of sedimentary formations, extending from the Barremian stage of the Cretaceous period at the base up to Quaternary deposits at the surface. The Lower Sahara actually represents a broad infill plain that has gradually subsided from the Lower Cretaceous through to the Quaternary. Indeed, this basin is primarily filled with post-Eocene continental Tertiary sediments, mainly conglomeratic sands interbedded with clay layers and clayey-sandy beds. Finally, the Neogene formations, originating mainly from the erosion of the Atlas Mountains, entirely conceal the underlying folded geological structures^35–37^, as show in Fig. 3.

The lithostratigraphic column of the Ouled Djellal region includes, from the oldest to the most recent: alterations of gray or white limestones and marls, sometimes gypsiferous, from the Lower and Upper Senonian. Next are lagoonal deposits and dolomitic, locally saline passages from the Middle Eocene (Lutetian), sandy and clayey levels with pebbles characterizing the terminal complex of the Miocene-Pliocene. Finally, there are accumulations of gravel, sand, and clay; filling of basins and wadi corridors from the Quaternary. The Quaternary terrains, widely represented, are the foundations of the current soils and the main local aquifers, particularly in the sectors of Wadi Djedi^36,38^, as seen in Fig. 2.

The Ouled Djellal region is underlain by a shallow Quaternary aquifer hosted in unconsolidated alluvial deposits that fill the valley of Wadi Djedi (Fig. 3). Groundwater recharge occurs primarily through infiltration from ephemeral flood flows and infiltration along the wadi beds after rainfall events. The aquifer, characterized by intergranular porosity, presents heterogeneous lithological facies composed mainly of sands, gravels, and silty layers, which locally control its hydraulic behavior. Its thickness is variable, reaching several tens of meters within the central valley. The piezometric surface follows the morphological axis of Wadi Djedi, indicating a flow direction from the piedmont towards the downstream plain with a gentle hydraulic gradient of about 4.3 × 10⁻². Pumping tests indicate a transmissivity on the order of 1.2 × 10⁻³ m²/s, consistent with moderate yield capacities from shallow boreholes ranging between 1.2 and 10 L/s. Although this aquifer is not highly productive, it constitutes the principal groundwater reservoir in the region, meeting domestic and agricultural demands. Nevertheless, the Quaternary aquifer remains sensitive to water-level declines due to limited natural recharge in this arid environment and the high extraction pressure during irrigation periods.

### Analysis and sampling of water

Twenty-three Groundwater (GW) samples from the study region were taken from boreholes representing the shallow alluvial aquifer of Ouled Djellal (Fig. 1). Approximately 1.5 dm^3^ polyethylene bottles have been used to store non-acidified water. Next, samples were kept at 4 °C and their chemical composition was determined by studying the components of Ca^+ 2^, Na^+^, K^+^, Mg^+ 2^, HCO_3_^-^, SO_4_^2-^, and NO_3_^-^, and Cl^-^. The standard analytical methods were used to analyze water at the hydrochemistry laboratory of Constantine University in Algeria. Titration method with the methyl orange end point was conducted for the determination of the bicarbonates. The chloride level was estimated using the Mohr technique of titrating and precipitating AgCl until silver chromate appears^39^. The nephelometric technique was used to determine sulfate^40^, while colorimetric analysis was used to examine nitrates^41^. A complex metric titration was used to determine the amounts of calcium and magnesium. To measure the sodium and potassium concentrations, emission spectrometry was utilized^39^.

Twenty-three groundwater samples were collected from electrically pumped boreholes at the different locations in the study area, representing the shallow alluvial aquifer of Ouled Djellal (Fig. 1). Following a 15-minute pumping period to remove any stored groundwater, samples were taken using two acid-washed polypropylene (PP) bottles, following the American Public Health Association’s protocol^42^. Using cellulose acetate 0.45 m filters, each sample was quickly filtered locally. Before being acidified to a pH of 2 with 5 mL of 6 N HNO_3_, the filtrate was immediately transferred to 1.5 L polyethylene (PE) bottles for the cation analysis. Without acidification, samples were transferred to 1.5-litre plastic bottles for anion analysis. Prior to being transported to the lab for analysis, our samples were maintained at a temperature of around 4 °C in an ice chest. Major ions analyzed included Ca²⁺, Mg²⁺, Na⁺, K⁺, HCO_3_⁻, SO_4_²⁻, Cl⁻ and NO_3_⁻, with all concentrations expressed in mg/L. At the location, the physicochemical parameters (electric conductivity, temperature, TDS, and pH) were measured with a WTW multiparameter (P3 MultiLine pH/LF-SET) made in Germany. With its conductivity cell and pH/redox electrode connected in parallel, the WTW Multi-Line P3/LF-SET multi-parameter meter can measure all three parameters simultaneously. It also has a linear and non-linear temperature compensation function, so it can be used with both natural and ultra-pure waters.

Chemical analyses were carried out at the Water Control and Quality Laboratory of the Biskra Unit of the Algerian Water Company (ADE Biskra), Algeria. Bicarbonate concentrations were determined in the field after sampling by titration with H_2_SO_4_ using methyl orange as the endpoint. In the laboratory, chloride was measured by the Mohr method (argentometric titration until the appearance of silver chromate). Sulphate was quantified using the nephelometric method, while nitrate was analysed by colorimetric analysis. Calcium and magnesium concentrations were determined by complexometric titration with EDTA. Sodium and potassium were measured by flame emission spectrometry. The TDS was estimated by weighing and drying at 103–105 °C in an oven. This was achieved by applying standard methods of the APHA 2017. Quality assurance included duplicate analyses of 10% of samples, regular calibration with certified reference standards, and blank analyses. To check the correctness of the analysis, the ionic balance was used with charge balance errors maintained within ± 5%, ensuring data reliability and analytical accuracy.

### Multivariate statistical techniques and data analysis

#### Cluster analysis (CA)

It is a technique that identifies the distinctive characteristics of each group by grouping enormous datasets from each entity into various clusters^43–45^. It is commonly used to classify hydrogeochemical processes in Groundwater by dividing collected water samples into important geological and hydrogeological categories, particularly for hydrochemistry investigations^43,46^. The cluster dendrogram was applied to offer a visual representation of the clustering activities by greatly showing the groupings and their proximity while reducing the complexity of the original data.

#### Principal component analysis (PCA)

It is known as a linear structure with complex multivariate datasets methods that may be efficiently statistically analyzed without scarifying information^47^. The quantity of variables can be reduced while maintaining the same degree of related variability^48^. At the end, the PCA remains an effective tool for a better understanding of the relationships between information about basic, indirectly observable features.

### Indexing approach

#### Chloro-alkaline indices

CAI-I and CAI-II were used to show the relationship and the origin and between the major elements such as SO_4_^2-^ versus (Ca^2+^; Mg^2+^), (HCO_3_^-^+ SO_4_^2-^) versus (Ca^2+^+Mg^2+^), (Mg^2+^/Ca^2+^) versus (Mg^2+^/Na^+^), (HCO_3_^-^) versus (Ca^2+^+Mg^2+^). They were applied to determine the ion exchange mechanism between GW and aquifer minerals (Eqs. 1 and 2)^49^.

1 $$\:\mathrm{C}\mathrm{A}\mathrm{I}-\mathrm{I}=\frac{{\mathrm{C}\mathrm{l}}^{-}-\:({\mathrm{N}\mathrm{a}}^{+}+{\mathrm{K}}^{+})}{{\mathrm{C}\mathrm{l}}^{-}}$$

2 $$\:\mathrm{C}\mathrm{A}\mathrm{I}-\mathrm{I}\mathrm{I}=\frac{{\mathrm{C}\mathrm{l}}^{-}-\:\left({\mathrm{N}\mathrm{a}}^{+}+{\mathrm{K}}^{+}\right)}{{{\mathrm{S}\mathrm{O}}_{4}}^{2-}+{{\mathrm{H}\mathrm{C}\mathrm{O}}_{3}}^{-}+{{\mathrm{C}\mathrm{O}}_{3}}^{2-}+{{\mathrm{N}\mathrm{O}}_{3}}^{2-}}$$

#### Irrigation water quality indices

In this study, six indexes including IWQI, SAR, PS, SSP, RSC, and Na%, respectively, were calculated using the physicochemical parameters of the groundwater samples as given in Table 2.

**Table 2:** The IWQIs, formula, and its reference.

IWQIs^*^	Formula	Reference
IWQI	$\:\mathrm{I}\mathrm{W}\mathrm{Q}\mathrm{I}=\sum\:_{i=1}^{n}{Q}_{i}\times\:{W}_{i}$	^50^
SAR	$\:SAR=\:\left(\frac{{Na}^{+}}{\sqrt{\left({Ca}^{2+}+{Mg}^{2+}\right)/2}}\right)$	^51^
Na %	$\:\mathrm{N}\mathrm{a}\mathrm{\%}=\:\frac{\left({\mathrm{N}\mathrm{a}}^{+}+{\mathrm{K}}^{+}\right)}{\left({\mathrm{C}\mathrm{a}}^{2+}+{\mathrm{M}\mathrm{g}}^{2+}\right)+\left({\mathrm{N}\mathrm{a}}^{+}+{\mathrm{K}}^{+}\right)}\times\:100$	^52^
SSP	$\:SSP=\:\frac{{Na}^{+}}{{Ca}^{2+}+{Mg}^{2+}+{Na}^{+}}\times\:100$	^53^
PS	$\:{PS=\:Cl}^{-}+\:\left(\frac{{SO}_{4}^{2-}}{2}\right)$	^54^
RSC	$\:RSC=\:\left({HCO}_{3}^{-}+{CO}_{3}^{-}\right)-\left({Ca}^{2+}+{Mg}^{2+}\right)$	^53^

^*^IWQIs were determined in meq/L.

#### Irrigation water quality index

Equation 3 was used to apply a non-dimensional scale with a range of 0–100 to the relationship between variables (Na^+^, EC, SAR, Cl, and HCO_3_^−^), in order to calculate IWQI:

3 $$\:IWQI=\:\sum\limits_{i=1}^{n}{Q}_{i}\times\:{W}_{i}$$

Q_i_ indicates the quality measurement’s results within the tolerance ranges, and W_i_ indicates the weight of each parameter (as seen in Table 3).

4 $$\:{Q}_{i}={Q}_{max}-\left(\frac{\left[\left({X}_{ij}-{X}_{inf}\right)\times\:{Q}_{imap}\right]}{{X}_{amp}}\right)$$

where $\:{X}_{inf}$: correspond to the lower limit of the class, X_ij_: is the observed value (for each parameter), X_amp_: is the amplitude class that the parameter falls within, and Q_imap_ is the class amplitude. Equation 5 was used to obtain the values of W_i_:

5 $$\:{W}_{i}=\frac{\sum\:_{j=1}^{k}{F}_{j}{A}_{ij}}{\sum\:_{j=1}^{k}\sum\:_{i=1}^{n}{F}_{j}{A}_{ij}}$$

where $\:i$ is the number of physicochemical parameters chosen by the model (from 1 to n), $\:\mathrm{F}$ is the auto value of component 1, $\:\mathrm{j}$ is the number of selected factors by the model (from 1 to k). Finally, $\:\mathrm{A}$ = The substantially limited of parameter $\:\mathrm{i}$ by factor $\:\mathrm{j}$.

**Table 3:** The range of limit values of the parameters used in the computation of quality measurement.

Q_i_	SAR	EC (µs/cm)	HCO_3_^-^ (meq/L)	Na^+^ (meq/L)	Cl^-^ (meq/L)	HCO_3_^-^ (meq/L)
0–35	SAR > 2 or SAR ≥ 12	EC < 200 or EC ≥ 3000	HCO_3_ <1 or HCO3 ≥ 8.5	Na < 2 or SAR ≥ 9	Cl < 1 or Cl ≥ 10	HCO3 < 1 or HCO_3_≥8.5
35–60	6 ≤ EC < 12	1500 ≤ EC < 3000	4.5 ≤ HCO_3_ <8.5	6 ≤ Na < 9	7 ≤ Cl < 10	4.5 ≤ HCO_3_ <8.5
60–85	3 ≤ EC < 6	750 ≤ EC < 1500	1.5 ≤ HCO_3_<4.5	3 ≤ Na < 6	4 ≤ Cl < 7	1.5 ≤ HCO_3_<4.5
85–100	2 ≤ EC < 3	200 ≤ EC < 750	1 ≤ HCO_3_<1.5	2 ≤ Na < 3	1 ≤ Cl < 4	1 ≤ HCO_3_<1.5

### Datasets and artificial intelligence (AI) s approaches

In this study, model training and validation were carried out using representative data samples to ensure reliable performance evaluation. The dataset was randomly divided into 70% (training set) and 30% (validation set), which is a widely adopted practice in water quality modeling. As an example, some researchers employed a similar data split when training Deep Learning (DL) models for water quality index prediction^23^, reaching consistent performance across multiple evaluation metrics (such as R² and RMSE). In the same vein^24^, implemented random data partitioning to assess the generalization ability of multilayer perceptron (MLP) networks and regression models in WQI. There are also other recent studies, such as^32,55^ who have also adopted comparable ratios (like 70–30, 80–20, or 75–25) to maintain an effective balance between adequate training data and validation accuracy.

**Fig. 4:**
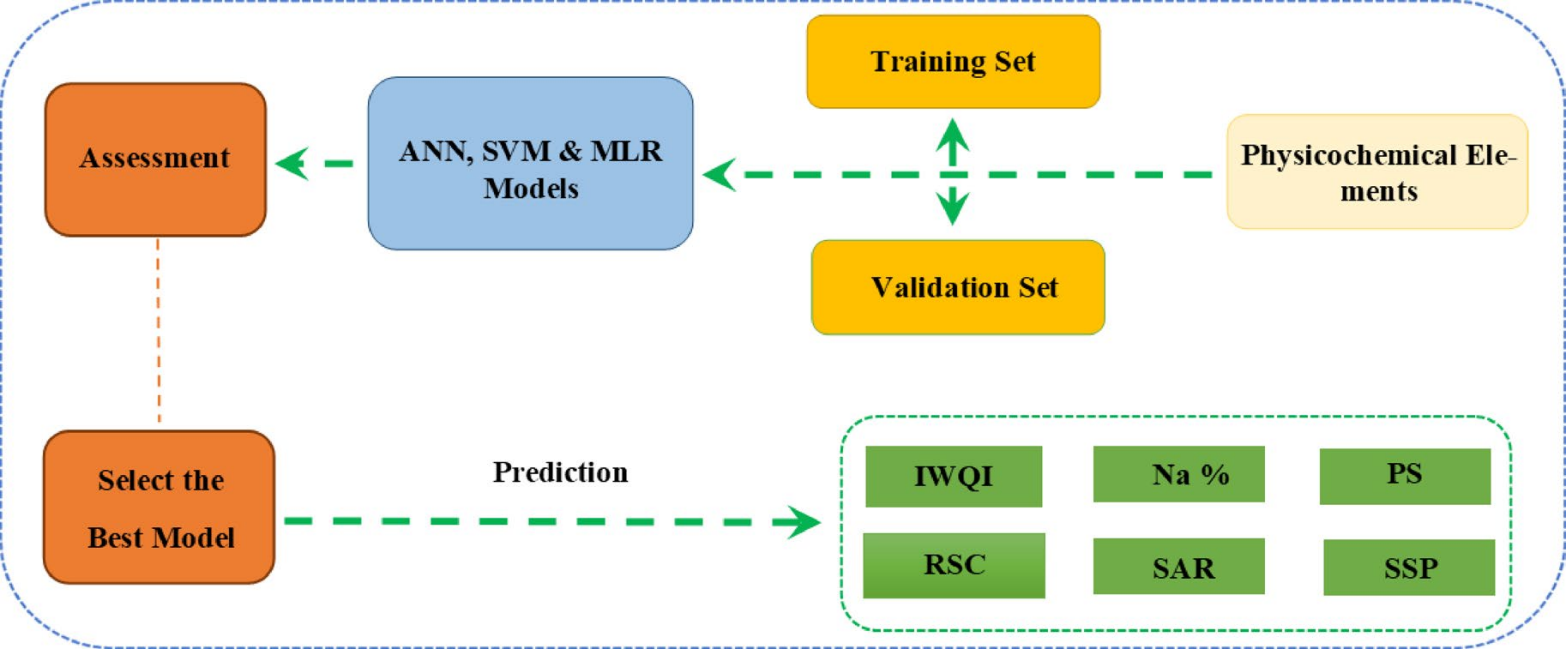
Flowchart for prediction of the IWQs using MLR, ANN, and SVM.

In our case, three models: multiple linear regression (MLR), artificial neural network (ANN), and support vector machine (SVM), were trained and evaluated (Fig. 4). We adopted this procedure in order to reduce the risk of overfitting and to ensure a reliable assessment of each model’s predictive performance. All data preparation, modeling, and analysis tasks were performed using SAS JMP Pro 18 software. The computations were carried out on a computer equipped with an Intel Core i7-3630 QM CPU (2.4 GHz) and a RAM of 8 GB.

#### Artificial neural networks (ANNs)

Artificial intelligence (AI) approaches have recently been acknowledged as powerful methods for modeling complex nonlinear phenomena in hydrology^56^. As an example, since 1980 s, ANNs have emerged as highly effective tools specifically to predict water quality^57^. These models consist of interconnected layers, input, hidden, and output with non-linear activation functions such as logistic, tanh, or ReLU, which allow them to uncover hidden patterns in water data without depending on predefined mechanistic rules^33,58–60^. A recent review indicated that ANN architectures are well-suited to a wide range of aquatic systems, from rivers and lakes to reservoirs and wastewater treatment plants, and excel at forecasting diverse water quality parameters^61^. Generally these models achieve a high predictive accuracy thank to their adaptability and minimal requirement for a priori assumptions, ANNs stand out as robust approaches for continuous monitoring and management of the quality of water^62^.

#### Support vector machine (SVM)

SVM models are a highly effective ML technique, specifically for assessing water quality, due to their robust generalization ability and resilience against overfitting^63,64^. SVMs are valuable for both classification and regression tasks, especially in environmental science. Some studies have demonstrated that SVMs can predict key water quality parameters with high accuracy^65^, while another study presented the fact that the SVM model accurately estimate the water quality factors with minor prediction errors, confirming its efficacy and performance^66^. In addition, hybrid models integrating SVM with WQI have shown strong predictive performance, achieving high classification accuracies as it was the case in groundwater quality assessment of the Egyptian Delta^67^, same thing for^68^, where SVM was also the most accurate algorithm for water quality classification prediction in India. Thus, SVM remains a good choice and has excellent advantages in dealing with small samples and complex nonlinear model problems. In comparison with other algorithms, it has advantages such as fast learning speed and strong generalization ability^69,70^.

#### Multiple linear regression (MLR)

MLR remains a key method across various fields due to its computational efficiency, interpretability and also its well-established theoretical framework^71^. Its capacity to quantify relationships between a dependent variable and multiple predictors facilitates robust predictive modeling while controlling for confounding factors. Recent studies still use MLR, such as^72^who added a brute-force approach^73^. also used MLR to evaluate water quality in Hanyuan Lake (China). In recent years, with advancements in machine learning (ML) models, MLR appear to be less frequently employed in some fields, in favor of methods such as ANN, SVM, random forests (RF), or XGBoost. Furthermore, the results concerning the efficacy of MLR are mixed, for example^74^, found that MLR were less accurate than ANN in predicting water quality parameters in Iran, while^75^ and^76^ demonstrated that both MLR and ML models were very close and had a high accuracy in water quality evaluation in Syria and Turkey, respectively.

### Evaluation of model performance and sensitivity analysis

To investigate the effect of model architecture on predictive accuracy, hyperparameter sensitivity analyses were conducted for three models (SVM, ANN, and MLR). For the SVM model, a linear kernel was applied, and the cost parameter (C) was varied between 0.01 and 5.0 across 20 independent trials. The optimal configuration was determined using the highest coefficient of determination (R²), the lowest Root Mean Square Error (RMSE), and Mean Absolute Error (MAE) values. While the ANN architecture comprised between one (1) and three (3) hidden layers incorporating multiple activation functions (tanh, sigmoid, and linear), to enhance nonlinear learning and generalization capacity. The tanh and sigmoid functions were primarily employed in the hidden layers to capture complex nonlinear relationships, while a linear activation function was used in the output layer to generate continuous index values. The network was trained with a learning rate of 0.1, using 12 input features (physicochemical parameters) and a single output neuron corresponding to the target variable (indexes such as SAR, IWQI, SSP, Na%, RSC, or PS). Model weights were optimized through the backpropagation algorithm to minimize the Mean Squared Error (MSE). This structure mirrors practices seen in recent WQI studies, such as^23,24^ where model hyperparameters were systematically tuned to enhance model robustness and generalization. The efficiency of the regression model was calculated using the statistical metrics root mean square error ($\:RMSE$), according to formula n° 6 and coefficient of determination ($\:{R}^{2}$), according to formula n° 7. Both indices are calculated as follow^75,76^:

Root Mean Square Error (RMSE)

6 $$\:RMSE=\:\sqrt{\frac{1}{N}{\sum\:}_{i=1}^{N}{({F}_{\:act}\:-\:{F}_{\:p})}^{2}\:}$$

Determination Coefficient (R^2^)

7 $$\:{R}^{2}=\:\frac{\sum\:{({F}_{\:act}\:-\:{F}_{\:p})}^{2}}{\sum\:{({F}_{\:act}\:-\:{F}_{\:ave})}^{2}}$$

It is possible to explain all parameters by: *F*_*p*_ which is the calculated or the simulated value, *N* stands for data entries total number, while $\:{F}_{\:act}$ stands for the actual real value that is based on laboratory analysis, and finally, *F*_*ave*_ stands for the mean value.

### Data processing, analysis, and Spatial mapping

The software Statistical Package for the Social Sciences “SPSS” (version 22) has been employed to perform statistical analyses on IWQIs and the physicochemical characteristics. Piper’s diagram was utilized to determine water types and assess hydrogeochemical evolution based on the composition of cations and anions. The PCA and CA were applied using the software Statistica (v.8) to determine the key constituents of GW and to enhance water quality assessments by simplifying data analysis into recognizable patterns. Then, spatial distribution maps of the groundwater quality indices were generated using the Inverse Distance Weighted (IDW) interpolation and Empirical Bayesian Regression Kriging (EBRK) methods using ArcGIS Pro (v 3.0.1). In order to assess the reliability of the interpolation results and minimize potential bias arising from the spatial distribution of sampling wells, cross-validation was performed using the leave-one-out approach. The validation included the computation of statistical performance indicators such as the Mean Error (ME), Root Mean Square Error (RMSE), Mean Absolute Error (MAE), and the Coefficient of Determination (R²). These metrics were used to compare the performance of IDW^2^ and EBRK, where lower RMSE and MAE and higher R² values indicated better model accuracy and prediction reliability. Finally, Multiple Linear Regression (MLR), Artificial Neural Network (ANN), and Support Vector Machine (SVM) models were computed using JMP 18 Pro (JMP, Cary, NC, USA).

## Results and discussion

### Hydrochemical properties of groundwater

Twenty-three GW samples from Ouled Djellal area were collected and subjected to analysis. They were studied experimentally with chemometric techniques. Water quality index and statistical methods like correlation analysis, principal component analysis, cluster analysis, and GIS techniques were used to study the hydrochemical parameters. Table 4 showed a descriptive summary of the parameters of the analyzed groundwater samples, which presented alongside the Food and Agriculture Organization (FAO) standards^77^. Below, the spatial patterns of major ionic constituents (Ca^2+^, Mg^2+^, Na^+^, K^+^, HCO_3_^−^, SO_4_^2−^, Cl^−^, NO_3_^−^) in the study area’s groundwater samples are discussed. The statistical analysis of the twenty-three groundwater samples (Table 4) shows that the hydrochemical parameters vary a lot. This is because of both natural geochemical processes and human activities. For several major parameters recommended by the FAO, the levels are well above the standards. The total hardness (TH) reaches very high levels (mean of 4950 °F), confirming the high levels of calcium and magnesium ions.

**Table 4:** Summary of descriptive results of groundwater samples in the study area.

Parameter^*^	Min	Max	Mean	SD	Coef. of variation (CV, %)	FAO*
pH	7.16	7.86	7.48	0.22	0.03	6.0–8.5
EC	2566	16,100	6994.61	3032.66	0.43	3000
TDS	1283	8050	3497.74	1515.93	0.43	2000
TH	890	1952.61	4950	871	0.18	-
Ca^+ 2^	232	701	469.09	137.17	0.29	400
Na^+^	60	1002	254.74	228.38	0.9	920
K^+^	5	70	21.57	15.21	0.71	2
Mg^+ 2^	75	780	194.65	156.05	0.80	60
SO_4_^−2^	207	560	343.74	91.74	0.27	960
Cl^−^	425	4467	1126.52	877.26	0.78	1065
HCO_3_^−^	195	800	473.52	137.48	0.29	610
NO_3_^−^	2.78	87	28.04	17.97	0.64	10

*Note: units are expressed in mg/L, except for TH (°F), pH (unitless), and finally, EC (µS/cm). “SD” for Standard Deviation.

*FAO: Food and Agricultural Organization [water Quality for agriculture, Laboratory determinations needed to evaluate common irrigation water quality problems].

In general, the distribution of calcium (Ca²⁺) showed a marked concentration in the northern part of the region, as indicated by sample 20, which presented darker tones on the spatial distribution map. These high concentrations (ranging from 232 to 701 mg/l, with an average of 469 mg/l) frequently exceed the FAO limit (400 mg/L) and primarily reflected the dissolution of carbonate minerals (calcite, dolomite) and evaporitic minerals (gypsum). For magnesium (Mg²⁺), it shows a distribution similar to calcium, with higher concentrations in the northern part of the plain. The concentrations range from 75 to 780 mg/l, with an average of 194.65 mg/l, well above the FAO standards (60 mg/l). This distribution reflected the alteration of magnesium minerals and cation exchange processes, particularly active in the clay soils of the Ouled Djellal region. As for the distribution of sodium (Na⁺), it revealed maximum concentrations localized in the center-north and at certain points in the center of the region, as illustrated by sample 20 with dark tones. The concentrations varied from 60 to 1002 mg/l, with an average of 254.74 mg/l, generally not exceeding the FAO threshold (920 mg/L), with a high coefficient of variation (*R* = 0.9) indicated strong spatial heterogeneity. This distribution reflected the accumulation of soluble salts through evaporation and the possible anthropogenic inputs related to irrigation return flows. Finally, potassium (K⁺) follows a distribution similar to sodium, with relatively high levels in the same geographical areas, although in lower concentrations (5 to 70 mg/l, with an average of 21.57), which clearly exceed the recommended value (2 mg/L), as shown in Table 4. This distribution indicated a mixed origin between silicate weathering and agricultural inputs. The analysis of the spatial distribution of cations in the Ouled Djellal aquifer revealed a strong geographical heterogeneity due to evaporation processes, which characterize arid regions^36,78–80^.

For the geographical distribution of anions, chlorides (Cl⁻) showed the highest concentrations in the northeast of the region, with extremely variable values (425 to 4467 mg/l, with an average of 1126.52 mg/L) exceeding the FAO recommended limit of 1065 mg/L, as seen in Table 4. As an example, sample 20 reflected a high concentration of Cl⁻. This distribution reflected the intense evaporation process, as well as possible anthropogenic influences (irrigation returns flow and domestic discharges). Sulfates (SO_4_²⁻) concentrations presented a significant quantities in the center of the region, which ranging from 207 to 560 mg/l and remaining below the FAO threshold (960 mg/L). This distribution is primarily due to the dissolution of gypsum in geological formations. Moreover, samples 21 and 18 suggest the highest concentrations of SO_4_²⁻. Bicarbonates (HCO_3_⁻) showed high levels in the north and northeast, which ranging from 195 to 800 mg/l in some samples, which exceed the FAO threshold value (610 mg/L), that confirm the significant influence of carbonate dissolution processes in hydrochemical evolution. Nitrates (NO_3_⁻) values varied from 2.78 to 87 mg/L (average 28.04 mg/L) and significantly exceed the FAO standard (10 mg/L) at several points, due to nitrogen fertilizers and agricultural effluents^81,82^.

The pH values revealed a remarkably homogeneous across the entire region (7.16 to 7.86 with an average of 7.48), that indicated a neutral to slightly alkaline environment, in line with the range recommended by the FAO for irrigation (6.0–8.5) and consistent with the carbonated nature of the geological substrate^81^. The EC values reached to 16,100 µS/cm with an average of about 6,995 µS/cm and the TDS reached to 8,050 mg/L with an average of about 3,498 mg/L, exceeding the FAO thresholds (3000 µS/cm and 2000 mg/L, respectively), which indicated a high mineralization that could affect the quality for agricultural use by posing salinity problems for soils and crops. This spatial distribution perfectly coincides with the accumulation zones of major cations and anions. The spatial analysis showed that the distribution of hydrochemical parameters in the shallow aquifer of Ouled Djellal is primarily controlled by evaporation processes in an arid context, with a preferential concentration of salts in the northern and central areas of the region. This spatial heterogeneity, resulting from the complex interaction between climatic, geological, and anthropogenic factors.

### Hydrogeochemical characteristics and water types

**Fig. 5:**
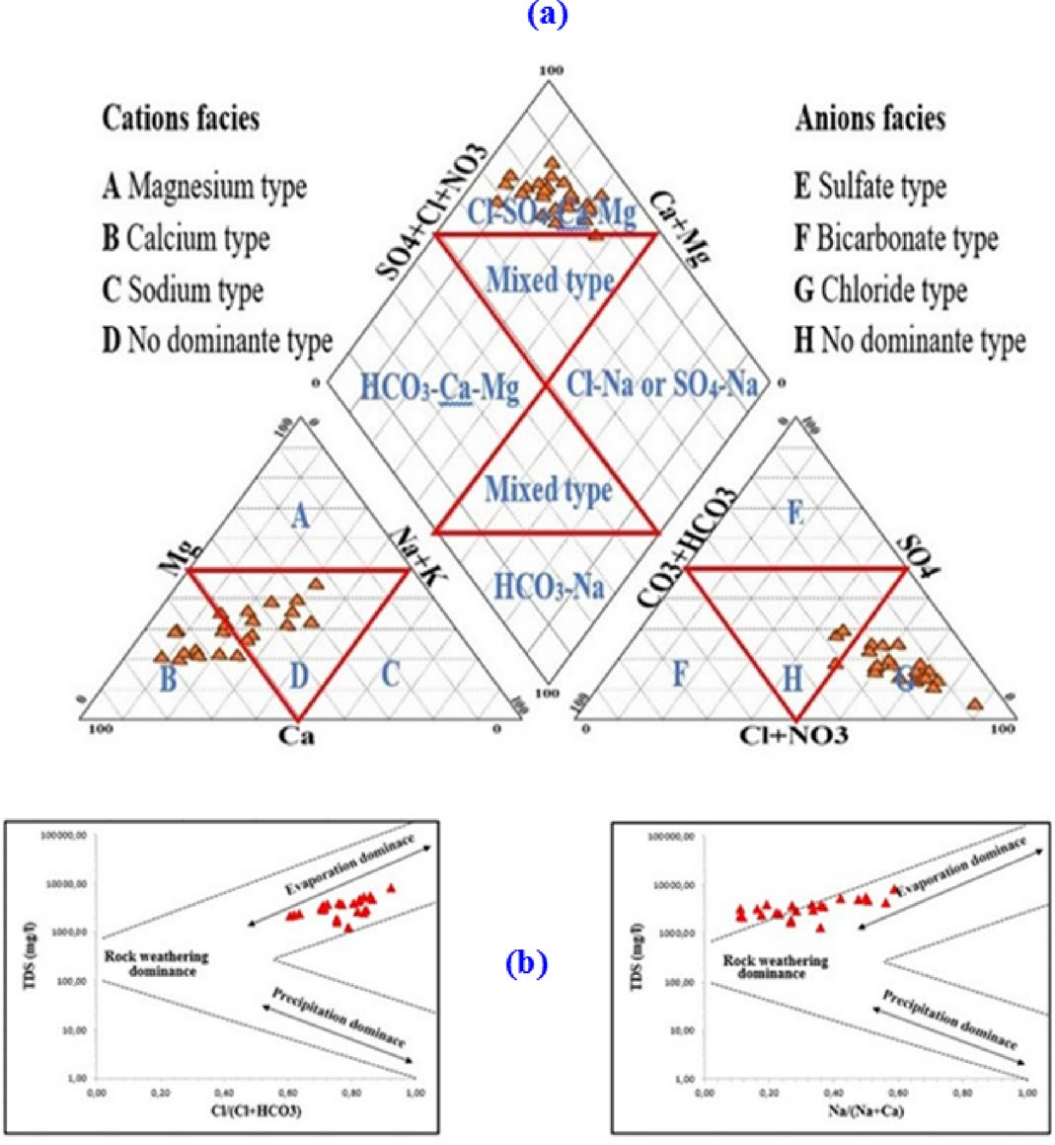
Hydrogeochemical characteristics and controlling mechanisms for the collected groundwater samples. (a) Piper diagram and (b) Gibbs diagram.

It is well established that GW chemistry is largely influenced by the type of rock formations that flows through and the duration of contact with those layers. As a result, the elements found in the water offer valuable insight into the characteristics of the aquifer that passes through^12,33^. Hydrochemical facies roughly assess the quantities of water that differ in their chemical composition, which is a combination of water-rock interactions through the rock matrix, solution kinetics, hydrogeological and environmental contexts, which is determined by calculating reaction quantities, considering ionic composition. Among the most widely used graphical methods for determining groundwater types are Piper and Gibbs diagrams^83,84^. The first one is a trilinear diagram that comprises a diamond and two equilateral triangles representing anions and cations respectively. Here, the diagram was constructed using Diagrams V 8.6 software. A single type of groundwater in Ouled Djellal was identified and classified according to their position on the diagram of Piper (Fig. 5). This group depended on the lithology of the area and the hydrodynamics of the water table. The cations fall within zones B and D, suggesting that the GW is mainly of the calcium type, with some samples showing no clear dominant cation. As for the anions, most of the samples are predominantly located in zone G, indicating a high prevalence of chloride. In the resulting diagram, all our samples are positioned in the upper zone, with Cl^−^, Ca^2+^ and Mg^2+^ being the main ions. An analysis of the molar concentrations of the different elements in the research area reveals the following trend among the cations: Ca^2+^> Mg^2+^> Na^+^> K^+^ evolving as follows: Cl^−^> SO_4_^2−^> HCO_3_^−^> NO_3_^−^. Thus, the groundwater type in Ouled Djellal aquifer revealed chloride water type for anions, and calcium and magnesium water type for cations, which classified as calcium-magnesium chloride water types due to the presence of saline, sulfate-rich evaporite formations, notably gypsiferous clays. The main ions distribution (Ca^2+^, Mg^2+^, Cl^−^ and SO_4_^2−^) is highly influenced by the lithology of the region and by anthropogenic activity, which leads us to think of an anthropogenic origin, like the quality of irrigation water, uncontrolled fertilization and domestic discharges from urban clusters dotted around this region.

### Processes influencing groundwater chemistry

**Fig. 6:**
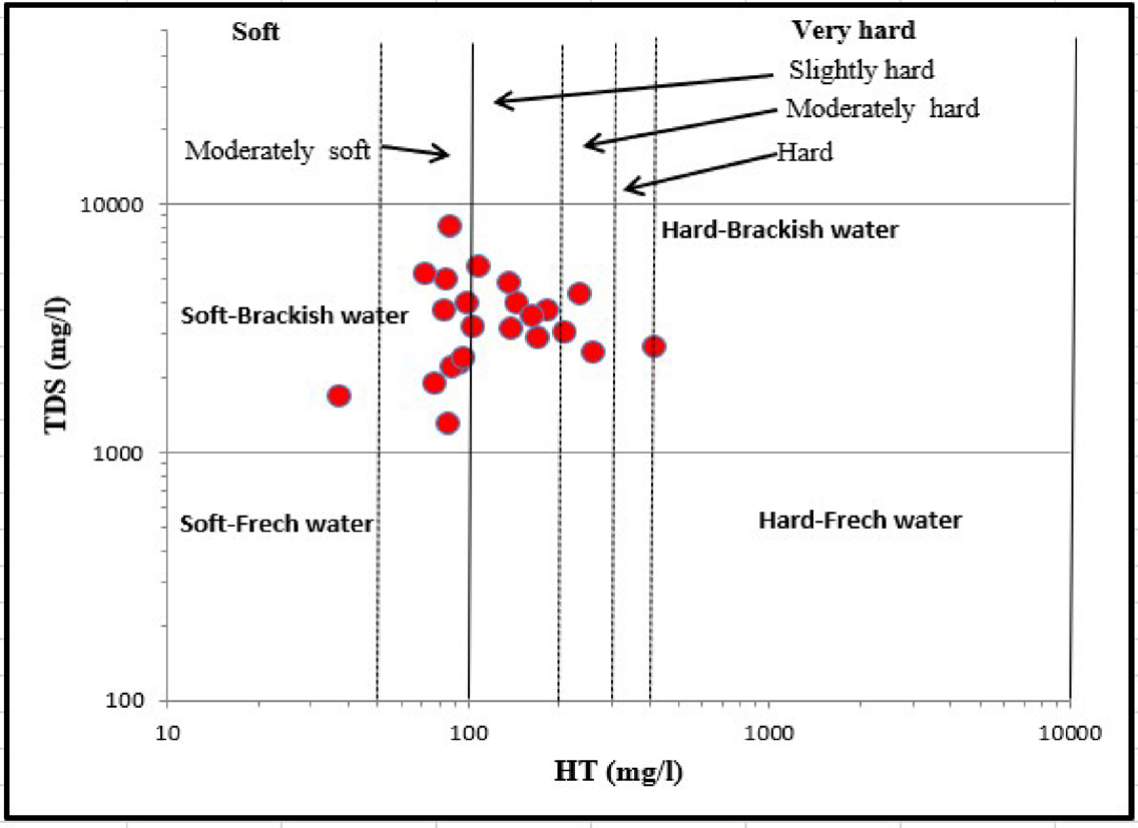
Plot of TDS Vs TH of groundwater samples.

The chemical composition of GW samples indicate that the impact of hydrochemical processes in the aquifer including evaporation and rock-water interaction^36,85,86^. Statistical analysis of the major ions (K^+^, Ca^2+^, Na^+^, Mg^2+^, SO_4_^2−^, HCO_3_^−^, Cl^−^, and NO_3_^−^) provides the fundamental basis for comprehending groundwater hydrogeochemical properties, which showed concentrations above the permissible limit for TDS and HT. Plotting of total dissolved salts (TDS) Vs. Total Hardness (TH). Figure 6 showed that 47% of GW samples were soft-brackish water and about 53% of samples were hard-brackish water. The interrelationships between anions and cations are a useful tool for governing mechanisms of GW chemistry.

#### Evaporation, soil-salt leaching and rock weathering

Gibbs diagram was used to interpret the changes in GW chemistry in the region of Ouled Djellal^84^. It classifies evolutionary processes into three types according to the dominance of rocks, precipitation and evaporation (Fig. 5). In our case, only one groundwater type was identified (soft-brackish water) in the study area. This group appears in the upper section of the diagrams, pointing to an evaporative origin and suggesting that evaporation is determining in shaping groundwater chemistry, while rock weathering has a minor influence. In fact, the Ouled Djellal area belongs has an arid climate with a high evaporation rate, which can explain the diversion of the water points towards the evaporation dominance zone (Fig. 5). This result is corroborated by the projection results on the Piper diagram, which indicating the importance of lithology on the chemical facies of GW in the studied region.

**Fig. 7:**
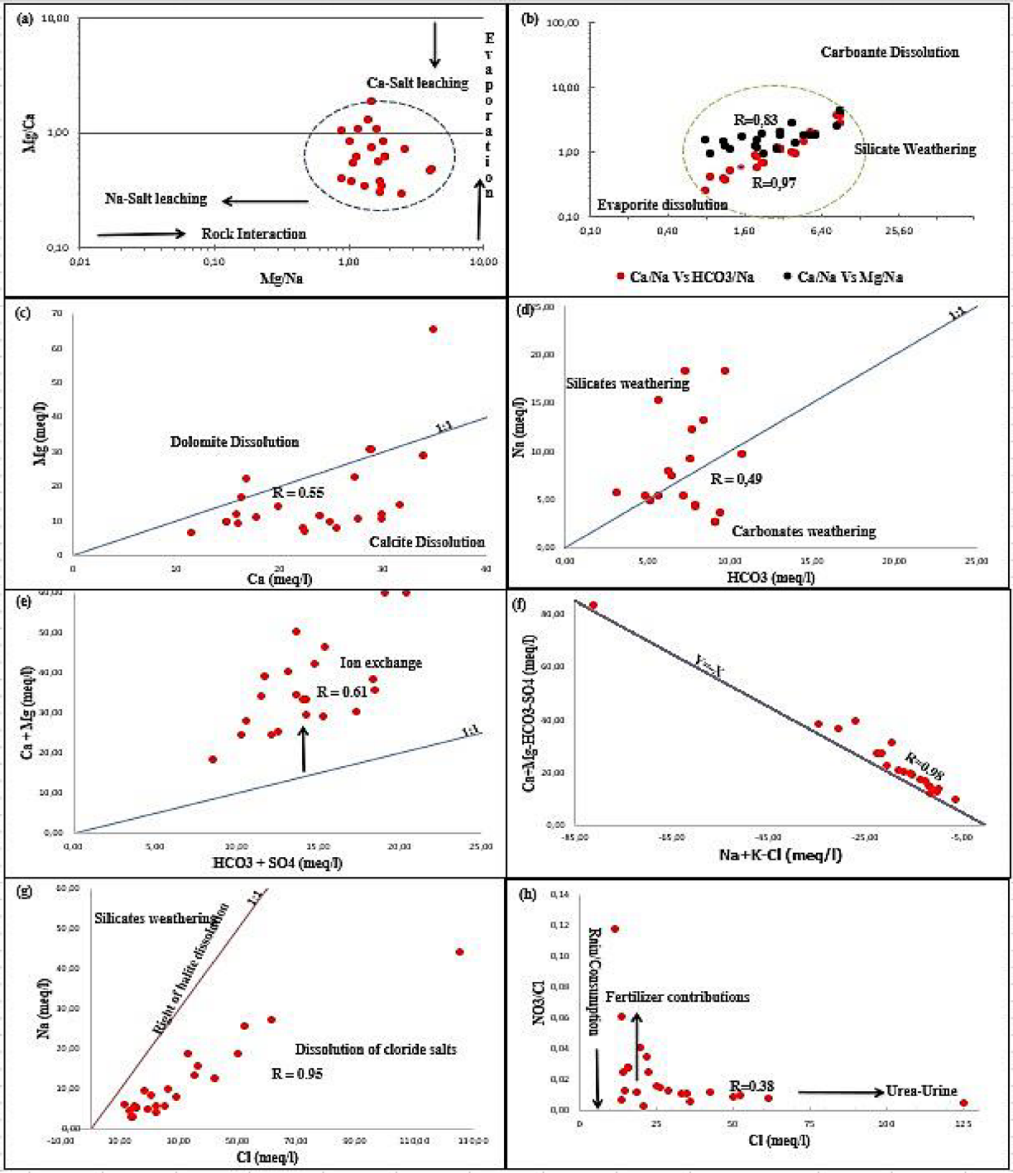
Stoichiometric relationships between major cations and anions of the groundwater samples in Ouled Djellal aquifer. **(a)** (Mg/Na and Mg/Ca), **(b)** ((Ca/Na) vs. (HCO3/Na) and (Ca/Na) vs. (Mg/Na)), **(c)** (Na Vs. Mg), **(d)** (HCO_3_ Vs. Mg), **(e)** ((HCO_3_ + SO_4_) Vs. (Ca + Mg)), **(f)** ((Ca + Mg - HCO_3_ - SO_4_) Vs. (Na + K - Cl)), **(g)** (Na Vs. Cl), **(h)** (NO₃/Cl Vs. Cl).

Based on Gibbs diagram, the ratio Ca^2+^/Na^+^ served as an indicator of salt leaching from soils. Nonetheless, the ratio Ca²⁺/Na⁺ may not be reliable in desert environments, as high evaporation rates and the abundance of sodium salts, commonly found in desert soils, which can elevate sodium levels relative to calcium due to water–rock and water–soil interactions^87,88^. As a result, Mg concentrations are not significantly affected by salt leaching from the soil or by saline precipitation during the early stages of evaporation. This makes Mg-to-cation ratios useful indicators of key processes in desert environments, especially the Mg²⁺/Na⁺ ratio for identifying salt leaching and the Mg²⁺/Ca²⁺ ratio for tracking evaporation. In this study, the Mg/cation plot (Fig. 7a), consistent with the Gibbs diagram, showed that the samples exhibit elevated Mg/Na and Mg/Ca ratios. These results suggest that evaporation was a key determinant in shaping groundwater chemistry, while water–rock interactions are likely a secondary factor. Our GW samples fall within the evaporation zone of the Gibbs diagrams, which indicating that the main components of the groundwater in the study area affected by intense evaporation, which leads to high TDS concentrations. In addition, the Gaillardet diagram^89^ was utilized to capture various hydrochemical reactions in unmixed conditions. The observed correlation (*R* = 0.509) between Na⁺ and Ca²⁺ indicated an exchange process between these ions in the groundwater flow^90^ (Fig. 7b). Therefore, the collected groundwater samples in Ouled Djellal aquifer were affected by silicates weathering and evaporation dissolution processes. The strong correlation between Ca^2+^/Na^+^ Vs. HCO_3_^−^/Na^+^, *R* = 0.97 and Ca^2+^/Na^+^ Vs. Mg^2+^/Na^+^, *R* = 0.83 in log-log space. Based on Fig. 7b, the composition of the GW is mainly governed by evaporation dissolution and silicate weathering.

The ratio Mg^2+^/Ca^2+^ indicated the dolomite and calcite dissolution processes. Mg^2+^/Ca^2+^ ratio > 1 also indicated the dolomite dissolution, while a ratio less than unity indicated the calcite dissolution^91,92^ in clay soils, due to ion exchanges between clay minerals and the solution, which lead to an enrichment of the water in calcium and released or adsorbed sodium according to local equilibrium conditions. The ion exchange processes can increase Ca^2+^ concentration with a proportional decrease of Na^+^^93^. In this study, GW samples have a ratio < 1, that reflecting the rapid dissolution of calcite relative to dolomite (Fig. 7c), and the predominance of Ca²⁺ in GW appears to derive from carbonate and evaporite mineral dissolution, with gypsum dissolution contributing as a secondary input.

Silicate formations are present in the clayey formation and siliceous sands, which widely found in the studied region of Ouled Djellal in the beds of wadis and the banks of temporary wadis. Their alteration and interaction with groundwater promote the gradual release of Na⁺ and Mg²⁺ ions^94,95^. A ratio HCO_3_^−^/Na^+^ > 1 suggested carbonates weathering, while reduced levels imply silicate-weathering dominance. Plotting the groundwater samples on the scatter plot of HCO_3_^−^ versus Na^+^ and in relation to slope line 1 (Fig. 7d), denoting a slight majority (52%) of the GW, which indicated silicates weathering processes. The scatter plot of (HCO_3_^−^ + SO_4_^2−^) versus (Ca^2+^ + Mg^2+^) was far above the slope line 1 (Fig. 7e), that highlighting the dominance of magnesium and calcium containing minerals. The observed mineralization can be linked to the weathering and dissolution of carbonate minerals, along with contributions from other sources of Mg²⁺ and Ca²⁺, such as gypsum or anorthite, or from changes due to cation exchange processes^91,96^.

#### Ions exchange processes

For understanding the hydrochemical reaction affecting on the groundwater quality, ion exchange in Eq. 8 and reverse ion exchange in Eq. 9 were applied^97^. These methods were used to determine the occurrence of cations exchange process, supported withthe relation between (Ca^2+^ + Mg^2+^ - HCO_3_^−^ - SO_4_^2−^) Vs. (Na^+^ + K^+^ - Cl^−^)^89^. If cation exchange significantly influences the ionic composition of groundwater, the relationship between these parameters is expected to be linear, with a slope of Y = –X. In this study, a strong linear alignment with the reference trend Y=-X. Figure 7f indicated an increase in Ca^2+^ + Mg^2+^ linked to a decrease in Na^+^ + K^+^ or probably an increase in HCO_3_^−^ + SO_4_^2+^. The strong correlation *R* = 0.98 between Ca^2+^ + Mg^2+^ - HCO_3_^−^ - SO_4_^2−^ & Na^+^ + K^+^ - Cl^−^, indicated that cations play a key role in regulating the hydrochemical composition of groundwater and the involvement of Ca²⁺, Mg²⁺, and Na⁺ in ion exchange reactions^98^, as represented by Eq. 8.

To analyze the ion exchange process, a plot of Na⁺ against Cl⁻ was used. Theoretically, halite dissolution should release equal amounts of Na⁺ and Cl⁻ as displayed in Eq. 10, and the concentrations of these ions in meteoric water infiltrating into groundwater are expected to be nearly equal. Therefore, halite dissolution regulates the concentrations of Na⁺ and Cl⁻ in the groundwater system, whereas sodium released from silicate weathering results in a broader Na⁺/Cl⁻ ratio^36,99^. Moreover, the scatter plot Na^+^ versus Cl^−^ in all samples was under the line of halite dissolution, indicate an excess of Cl^−^, due to other Cl origin (Fig. 7g). Finally, the elevated concentration of Cl⁻ in GW is mainly attributed to climatic factors like intense evaporation, but it may also result from anthropogenic sources like domestic wastewater and irrigation return flow.

8 $$\:2NaX+{Ca}^{2+}\to\:2{Na}^{+}+{CaX}_{2}$$

9 $$\:Ca{X}_{2}+2{Na}^{+}\to\:{Ca}^{2+}+2NaX$$

10 $$\:NaCl\to\:{Na}^{+}+{Cl}^{-}$$

#### Anthropogenic input

Chloride behavior conservatively in natural waters, as it is largely unaffected by chemical, biological, or physical processes. The NO_3_⁻/Cl⁻ ratio is a useful indicator of mixing processes or biological activity affecting nitrate and chloride in groundwater. Chemical fertilizers typically contain high levels of nitrogen and low amounts of chloride, whereas domestic and animal wastewaters tend to have high chloride concentrations and low NO_3_⁻/Cl⁻ ratios^100^.

Figure 7h showed the variations in the NO_3_^−^/Cl^−^ molar ratio as a function molar concentration of Cl^−^ in groundwater and different poles and potential vectors of nitrates confirm the agricultural origin for nitrates and domestic wastewater or the return of irrigation water for chlorides. The low correlation coefficient (*R* = 0.38) between NO_3_^−^/Cl^−^ and Cl^−^ concentration testifies to a different exogenous origin of nitrates and chlorides.

#### Chloro-alkaline index

**Fig. 8:**
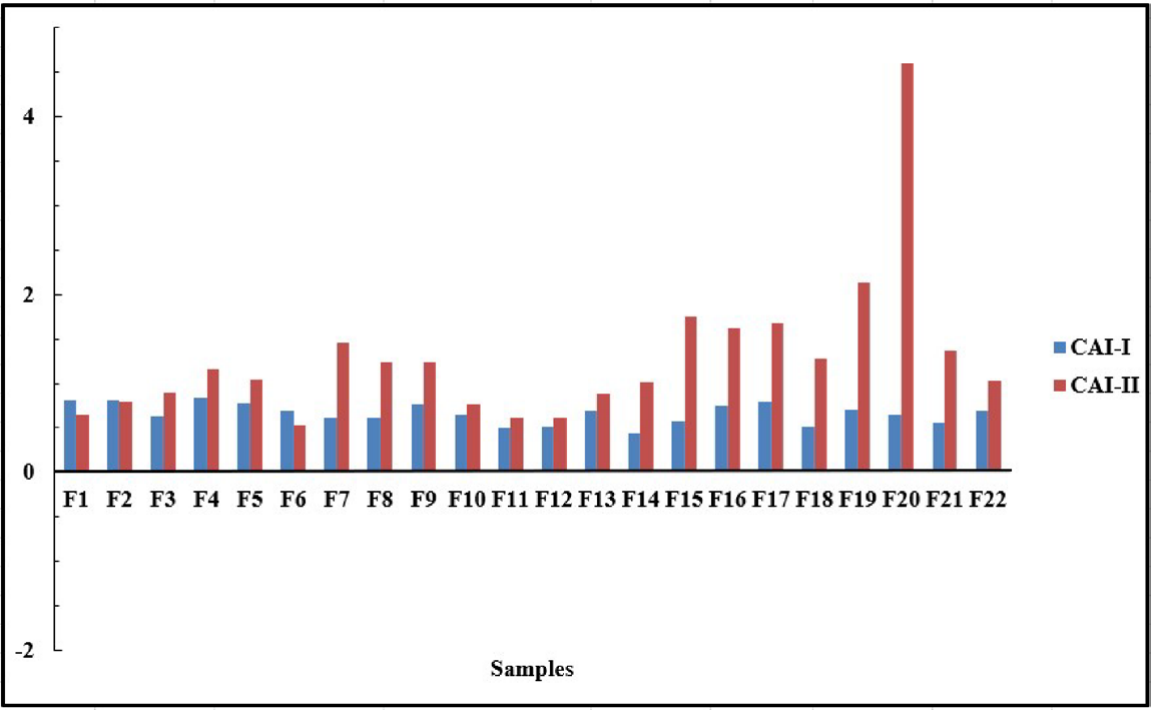
Cations exchange diagram for the groundwater in Ouled Djellal aquifer.

During groundwater flow, the water is exposed to certain formations that have the properties of exchanging their ions for those continuous in the water. The basic exchange index is the ratio between the ions exchanged and the ions of the same nature originally existing. Two basic exchange possibilities can occur: either the exchange of alkalis with alkaline earths (CAI-I), according to Eq. 1, or the exchange of alkaline earths in water with alkalis (CAI-II), according to Eq. 2. Generally, K+ & Na + in GW are exchanged with Ca^2+^ & Mg^2+^ in aquifer matrix, resulting in a direct ion exchange^101^. The GW samples showed a positive value for both indices (CAI-I & CAI-II) suggesting the contribution of direct ion exchange in the system (Fig. 8).

#### Geochemical modelling

Geochemical modelling was used to explain the mechanisms by which mineralization occurs. This was carried out by simulating natural evaporation using the Phreeqci software^102^, which computes aqueous speciation and thermodynamic equilibrium conditions of groundwater, considering the dominant mineral phases in the aquifer. Mineral reactions (dissolution or precipitation) are constrained by their respective saturation indices. Calculation of the saturation index (SI) of dissolved minerals in water has been carried out using Debye & Hückel’s law, as explained by^103^ and expressed as follows Eq. 11.

11 $$\:IS=\:\frac{Log\:PAI}{k}$$

when, IS > 0.5, water is supersaturated with respect to a mineral. This mineral would tend to precipitate, against when IS < −0.5, the water is undersaturated with respect to a mineral. This indicates the minerals tendency to dissolve.

**Fig. 9:**
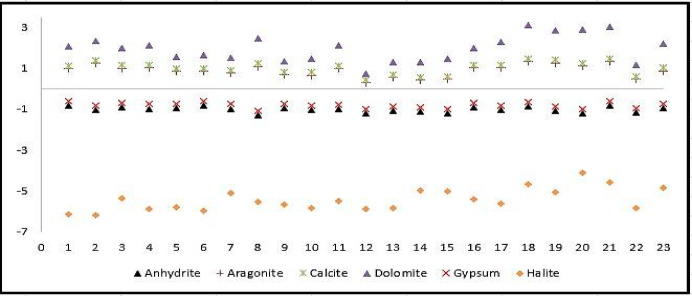
Saturation index for carbonates and evaporates in Ouled Djellal aquifer.

The calculated values of the SI for carbonates and evaporates elements are depicted in Table 5. The SI values were calculated for the carbonate elements such as, calcite, aragonite, and dolomite (Fig. 9). When the SI > 0.5, demonstrating that the GW was supersaturated with regard to these minerals, and precipitation could have occurred. The supersaturation of these elements may lead to a preferential precipitation, which could diminish the concentrations of Ca^2+^, Mg^2+^ and HCO_3_⁻ in groundwater due to high temperature, high evaporation rate, and low rainfall. On the other hand, the SI < 0.5 for gypsum, anhydrite, and halite, suggesting persistent under-saturation. Consequently, Na⁺, Cl⁻, SO_4_²⁻, and Ca²⁺ ions may continue to be released into groundwater through weathering of these minerals. The dissolution of these minerals was indicated by the high Na⁺ & Cl⁻ correlation (*R* = 0.95) and Ca²⁺ & SO_4_²⁻ correlation (*R* = 0.43).

**Table 5:** Descriptive results of the calculated SI in the study area.

Elements	Carbonates elements			Evaporates elements		
	Aragonite	Calcite	Dolomite	Anhydrite	Gypsum	Halite
Minimum	0.28	0.43	0.71	−1.30	−1.08	−6.18
Maximum	1.33	1.47	3.00	−0.83	−0.61	−4.11

### Statistical analysis

#### Elementary statistics and correlation analysis

**Fig. 10:**
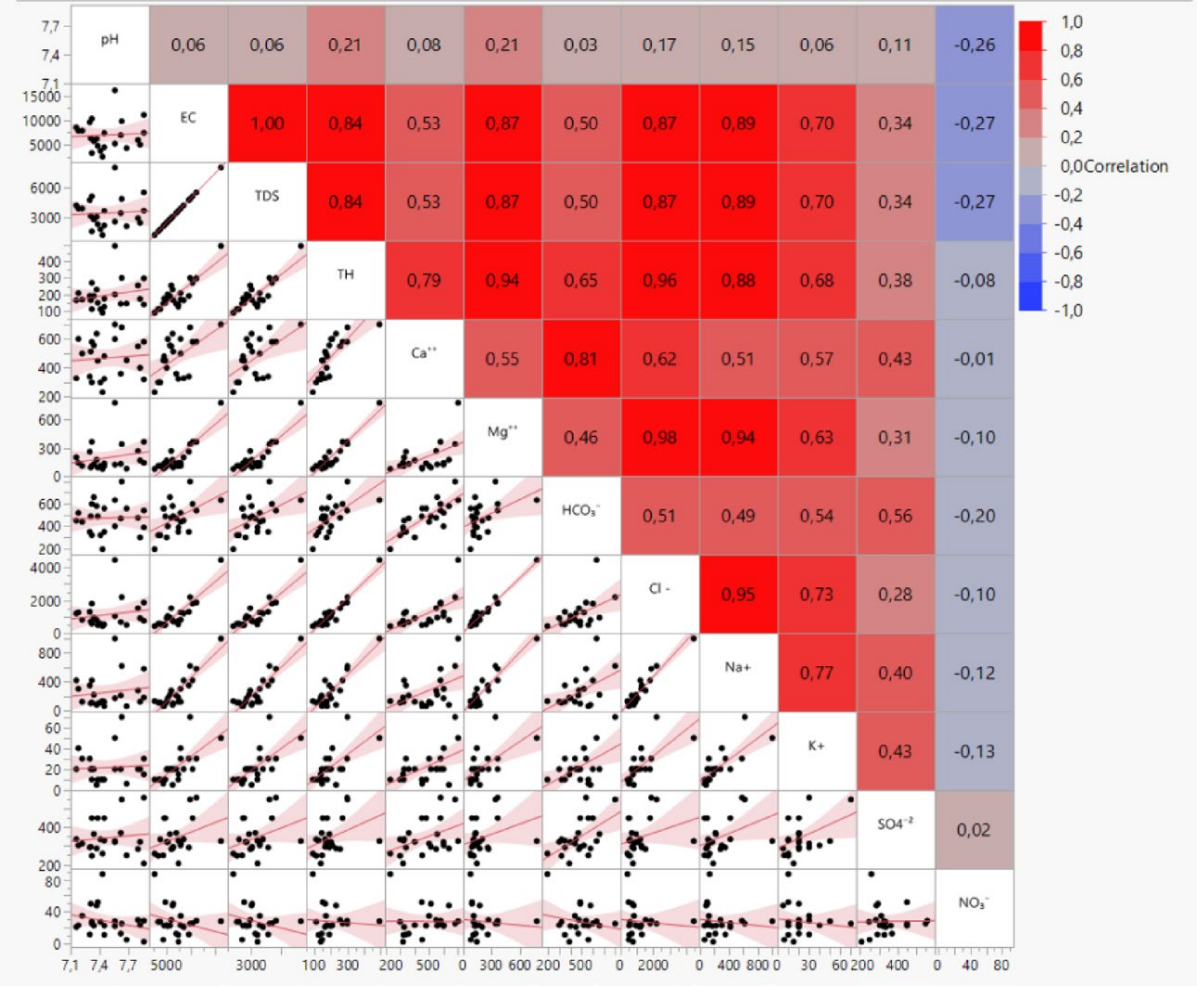
Scatterplot and correlation matrix for the groundwater in the Ouled Djellal aquifer.

The correlation matrix (Fig. 10) for the variables revealed a close relationship between the various elements, such as Na^+^, Cl⁻, Mg^2+^ K^+^, Ca^2+^ and HCO_3_^−^ which are significantly correlated with EC and TDS (0.502 < *R* < 0.894). These correlations indicated that water chemistry in the Ouled Djellal region controlled by lithological factors and various hydrogeochemical processes. These results demonstrate a strong influence of these elements on regional groundwater chemistry. Conversely, the lack of significant correlation between EC/TDS and SO₄²⁻/NO₃⁻ implies negligible involvement of these anions in salinization processes.

Significant positive correlation values were observed between all the elements except NO_3_⁻ and SO_4_^2^⁻. These trends resulting from leaching processes and dissolution of evaporite minerals. While chloride salts may release Cl⁻ into GW, while the high Na⁺ - Cl⁻ (0.954) and K⁺ - Cl⁻ (0.775) primarily indicated dissolution of evaporitic salts such as halite due to the influence of evaporation. A secondary contribution may arise from silicate alteration, which can affect the mobility of Na⁺ and K⁺ associated with Cl⁻ in certain lithological contexts^36^. According to the SO_4_^2^⁻ concentrations results (max 11.67 meq/L^− 1^) at borehole 18, the origin can be attributed to the limited dissolution of sulphur minerals (a trace of gypsum or locally oxidized pyrite), which found in limestone, sand. While the element NO_3_^−^ showed an insignificant negative correlation with the whole elements, which indicated an anthropogenic origin including agricultural practices. Strong correlations underline dissolution of gypsum, halite, and salts as the primary solute source, exception for HCO3⁻ (carbonate weathering) and NO₃⁻ (anthropogenic input). Figure 10 indicated that R value for Mg^2+^, Cl⁻, K^+^ and NO_3_⁻ in Ouled Djellal area was relatively high, due to more reactive of ions to hydrological, geological, and anthropic factors. The statistical results are fully consistent with the geochemical evidence, confirming that evaporation and saline mineral dissolution were the dominant mechanisms shaping GW composition in the shallow Oued Djellal aquifer. Minor discrepancies among certain ions (SO_4_²⁻ and NO_3_⁻) are clearly explained as resulting from localized geological or anthropogenic inputs.

#### Principal component analysis (PCA)

**Fig. 11:**
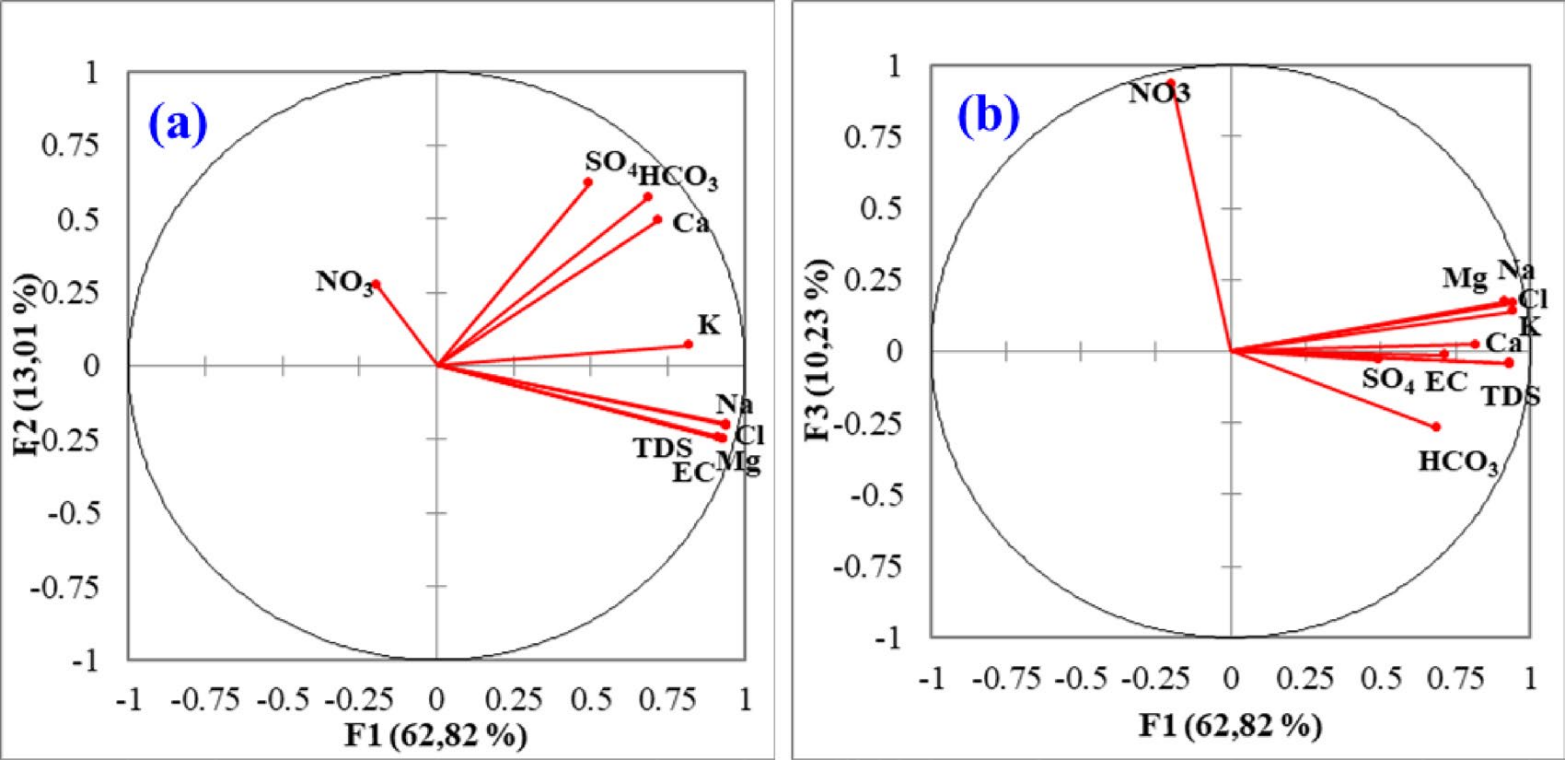
PCA scores: **(a)** F1 vs. F2 **(b)** and F1 vs. F3.

According to the PCA results, F1 accounts for 62.82% of the total variance, indicated a well-defined structure in the sampled data, which suggests a strong relationship among the key factors influencing sample composition. These relationships were further supported by the correlation matrix (Fig. 10), which revealed significant positive correlations between Na⁺, Cl⁻, Mg²⁺, K⁺, Ca²⁺, HCO_3_⁻, EC, and TDS (*r* = 0.89, 0.87, 0.87, 0.70, 0.52, and 0.50 (Fig. 10). Analysis of F1-F2 (Fig. 11a) showed that the F1 factor is strongly positive loading of EC, TDS, Na^+^, Cl⁻, Mg^2+^ and K^+^ and to a lesser extent by Ca^2+^, demonstrating that salinity originates from multiple sources, including evaporite dissolution, leaching of saline soils (containing clays, gypsum, and anhydrite), aqueous ion exchange processes, and anthropogenic contributions (Table 6).

At the same time, F2 explains 13.01% of the total variance of the data set and demonstrates lower positive loadings of SO_4_^2^⁻ and HCO_3_^−^ (0.385 and 0.329), respectively (Fig. 11a). These results indicated that SO_4_^2^⁻ and HCO_3_⁻not influence on water mineralization and predicts the different lithological origin. This likely results from carbonate weathering, which reflecting the impact of acid-base equilibrium on groundwater chemistry^104^ coupled with dissolution of sulfate minerals (notably gypsum) from the soil matrix. Regarding F1-F3, F3 explains 10.23% (Fig. 11b) and revealed a significant positive NO_3_^−^ load (0.869). Table 6 indicated the arrival of agricultural pollution or a mixture of deep water with surface water. This factor is associated with chemical inputs to the farm, mineralization of soils and non-agricultural sources or deep water mixing with surface water. Oxidation conditions promote the microbial oxidation of fertilizer NH_4_⁺ to NO_3_⁻ through nitrification processes^105^, as shown in the following reaction (Eq. 12).

12 $$\:{NH}_{4}^{+}\:+\:2{O}_{2}=\:{NO}_{3}\:+\:{H}_{2}O\:+\:2{H}^{+}$$

**Table 6:** Factor analysis of the variables.

Variable (unit)	F1	F2	F3
**EC** (µs/cm)	**0.866**	0.062	0.002
**TDS** (mg/l)	**0.866**	0.062	0.002
**Ca**^**2+**^ (mg/l)	**0.514**	0.245	0.000
**Mg**^**2+**^ (mg/l)	**0.835**	0.058	0.030
**HCO**_**3-**_**(**mg/l)	0.475	**0.329**	0.071
**Cl**^**-**^ (mg/l)	**0.886**	0.041	0.028
**Na**^**+**^ (mg/l)	**0.889**	0.039	0.019
**K**^**+**^ (mg/l)	**0.667**	0.005	0.001
**SO**_**4**_^**-2**^ (mg/l)	0.245	**0.385**	0.001
**NO**_**3**_^**-**^ (mg/l)	0.038	0.076	**0.869**

#### Cluster analysis (CA)

**Fig. 12:**
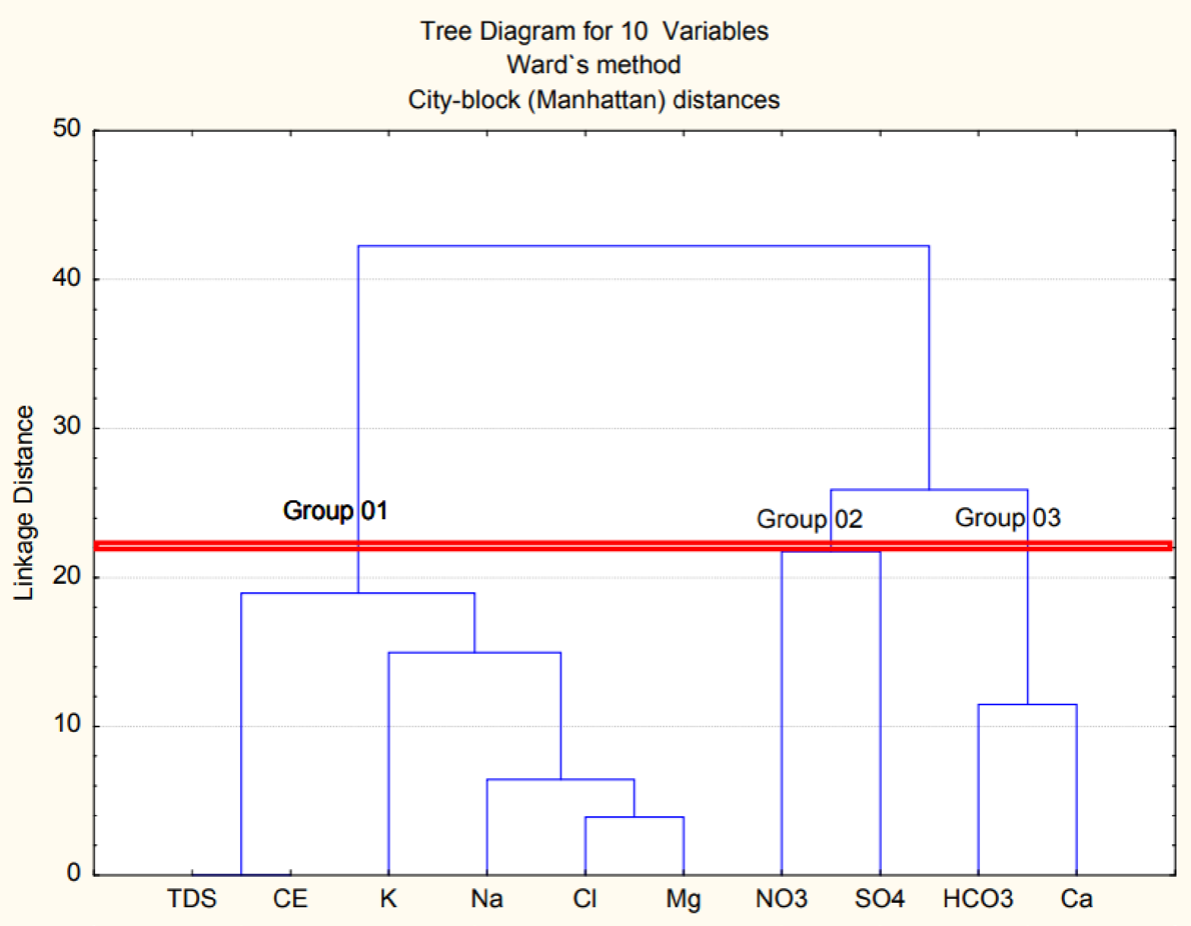
Cluster dendrogram for variables.

Hierarchical Ascending Classification (HAC) was applied using City-block (Manhattan) as the measurement distance between the analyzed GW samples. Ward’s technique was used as the linking rule for the classification of data acquired from water in Ouled Djellal. The results are presented in the form of a dendrogram of the 10 parameters, such as EC, TDS, Mg^2+^, K^+^, Ca^2+^, Na^+^, HCO_3_⁻, SO_4_^2^⁻, Cl⁻, and NO_3_⁻ (Fig. 12). Analysis of the diagram revealed three distinct statistical classes and indicated that TDS and EC may serve as key discriminators among elemental parameters (Fig. 12).

The first group indicated a strong correlation between Mg^2+^, Cl^−^, Na^+^ K^+^, EC and TDS, emphasizing the dominance of evaporitic elements in the GW chemistry of Ouled Djellal aquifer (Fig. 12). This evaporitic signature revealed Cl⁻- Ca²⁺ - Mg²⁺ facies. In contrast, Group 1 revealed a weak Mg²⁺-Ca²⁺ correlation, which indicative of Ca²⁺ dual origin from both carbonate and evaporitic sources. The second group showed a close association between NO_3_^−^ and SO_4_^2^⁻ and a dissociation of the other chemical elements present in the GW of Ouled Djellal aquifer, due to the anthropogenic origin of NO_3_⁻ and, lithological through the dissolution of gypsiferous clays and anthropogenic for SO_4_^2^⁻. Group 3 revealed an association between HCO_3_⁻ and Ca^2+^, which reflecting the same carbonate origin.

#### Suitability of water for irrigation

**Fig. 13:**
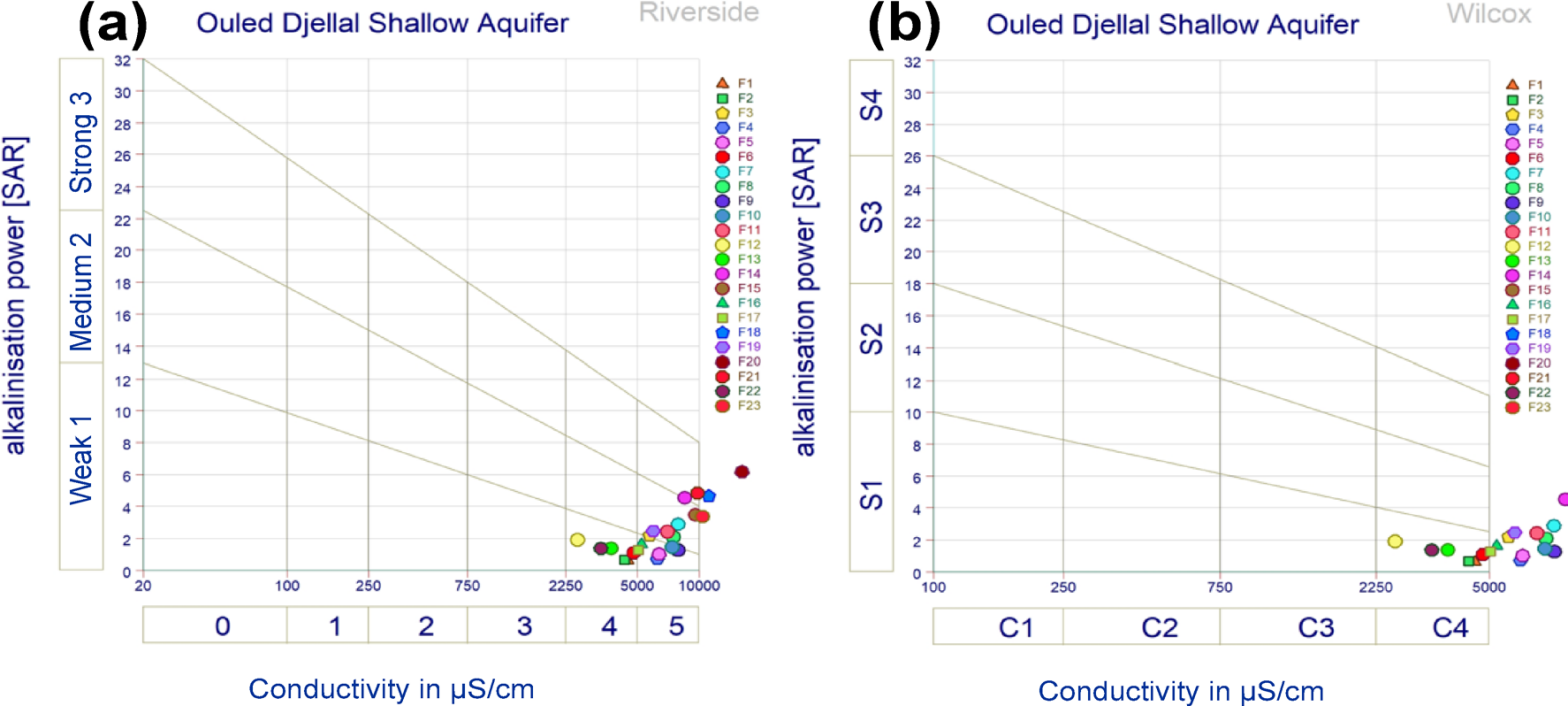
Riverside and Wilcox Diagrams.

More than 60% of the samples from the shallow Ouled Djallal aquifer showed a moderate to high risk of salinization associated with EC values exceeding 3000 µS/cm (Fig. 13a), which indicating a danger of soil alkalization and lead to clay dispersion and decrease permeability. The Wilcox diagram, also based on SAR and EC showed that most of the groundwater samples fall into the C3–C4/S1–S2 classes, which indicating excessive salinity (C4) (Fig. 13b) due to water-rock interactions, the dissolution of gypsum and halite, and irrigation return flow. The Gibbs diagram confirms the predominance of the evaporative process, while the ion exchange indices (CAI-I and CAI-II) revealed a direct Na⁺/Ca²⁺ exchange typical of areas with high agricultural activity. The majority of samples exceed the thresholds recommended by the FAO for irrigation (EC > 3000 µS/cm and often a high SAR), making rational management essential: tolerant crops, control of irrigation volumes, and regular monitoring of salinity and SAR. This high salinity limits agricultural use without proper management, restricts crop choices, and requires specific practices such as amendments, localized irrigation, or soil salinity monitoring.

**Fig. 14:**
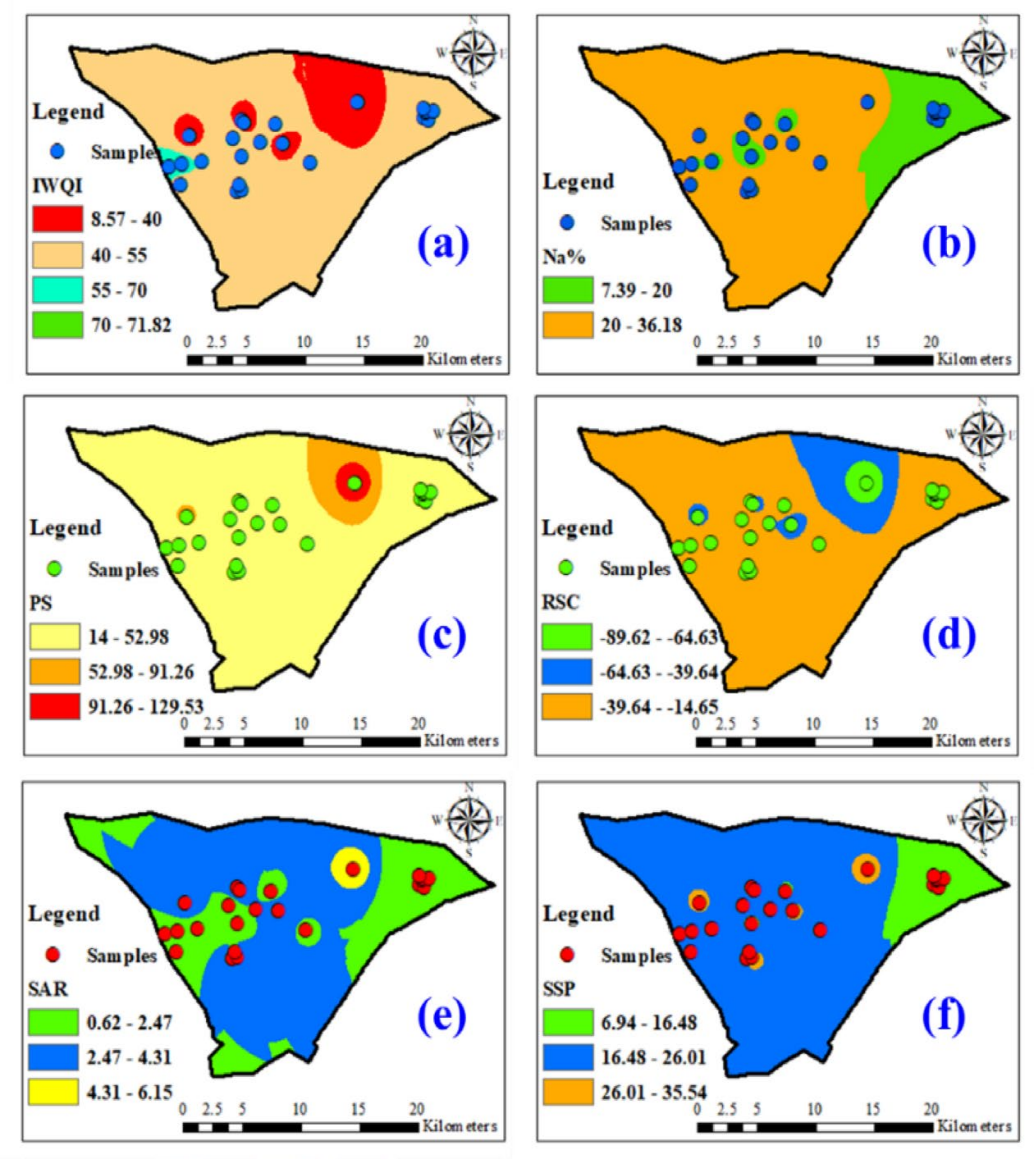
Irrigation water quality indices (IWQIs): **(a)** IWQI, **(b)** Na%, **(c)** PS, **(d)** RSC, **(e)** SAR, and **(f)** SSP. Map created using ArcGIS Pro 2.8.8 (Esri; https://www.esri.com/arcgis/about-arcgis).

According to Fig. 14, the spatial distribution of the IWQIs shows an area in the north and islands in the center, indicating lower water quality for irrigation in these sectors, hence the increased risk of soil salinization. The rest of these areas show more favorable indices. This distribution throughout the study region is graphically represented by the dataset analysis shown in Fig. 14(a), which also highlights the range of water quality values found. There is a significant variation in the water quality regulations, as seen by the IWQI readings, which range from 8 to 71 with an average of 39.5. The geospatial analysis identifies areas with water quality that may have an adverse effect on crop and soil health (Fig. 14(a)). In the region, a sizable percentage of water samples are classified as unsuitable for irrigation. This finding is significant because it raises concerns about potential negative effects on the soil’s capacity to retain moisture, distribute nutrients, and sustain crop yields overall. To counteract these consequences, eco-friendly water management strategies must be put into place. Regarding the percentage of sodium (Na%), the entire region, except for the northeastern center and the islands in the center, is characterized by high Na% values, suggesting an increased risk of sodicity, requiring amendments and modifications to agricultural practices to ensure good plant growth (Fig. 14b). Regarding soil permeability, the distribution reveals that reduced permeability mainly affects the northeast and, to a lesser extent, the center, correlated with areas of high sodicity (Fig. 14c). The distribution map of residual sodium carbonate revealed (RSC) indicated that the highest values were concentrated in the northeast and center, due to soluble carbonates in the soil at these depths (Fig. 14d). This may indicate a soil rich in limestone or marl characterized by a high carbonate content.

The sodium adsorption ratio, overall, is higher in the northeast, indicating the possible emergence of alkaline soils with reduced permeability, with a decreasing gradient towards the rest of the region (Fig. 14e). These suggest that GW in Ouled Djellal is generally suitable for irrigation. However, when saline water is used for irrigation, lower Sodium Adsorption Ratio (SAR) values are preferable. Sodium-related risks also depend on the total salt concentration in the irrigation water. Thus, water with salinity levels between 1.5 and 3.00 mS/cm and SAR > 4 should be applied cautiously to avoid soil degradation. It is important to note that soil samples must be taken annually to avoid potential salinity risks. The distribution map of soluble sodium percentage (SSP) revealed that the highest values were located in the northeast and center parts (Fig. 14f), overlapping with areas of a high SAR values (Fig. 14e), indicating problems of soluble sodium accumulation.

#### Spatial distribution maps supported with the EBKRP method

**Fig. 15:**
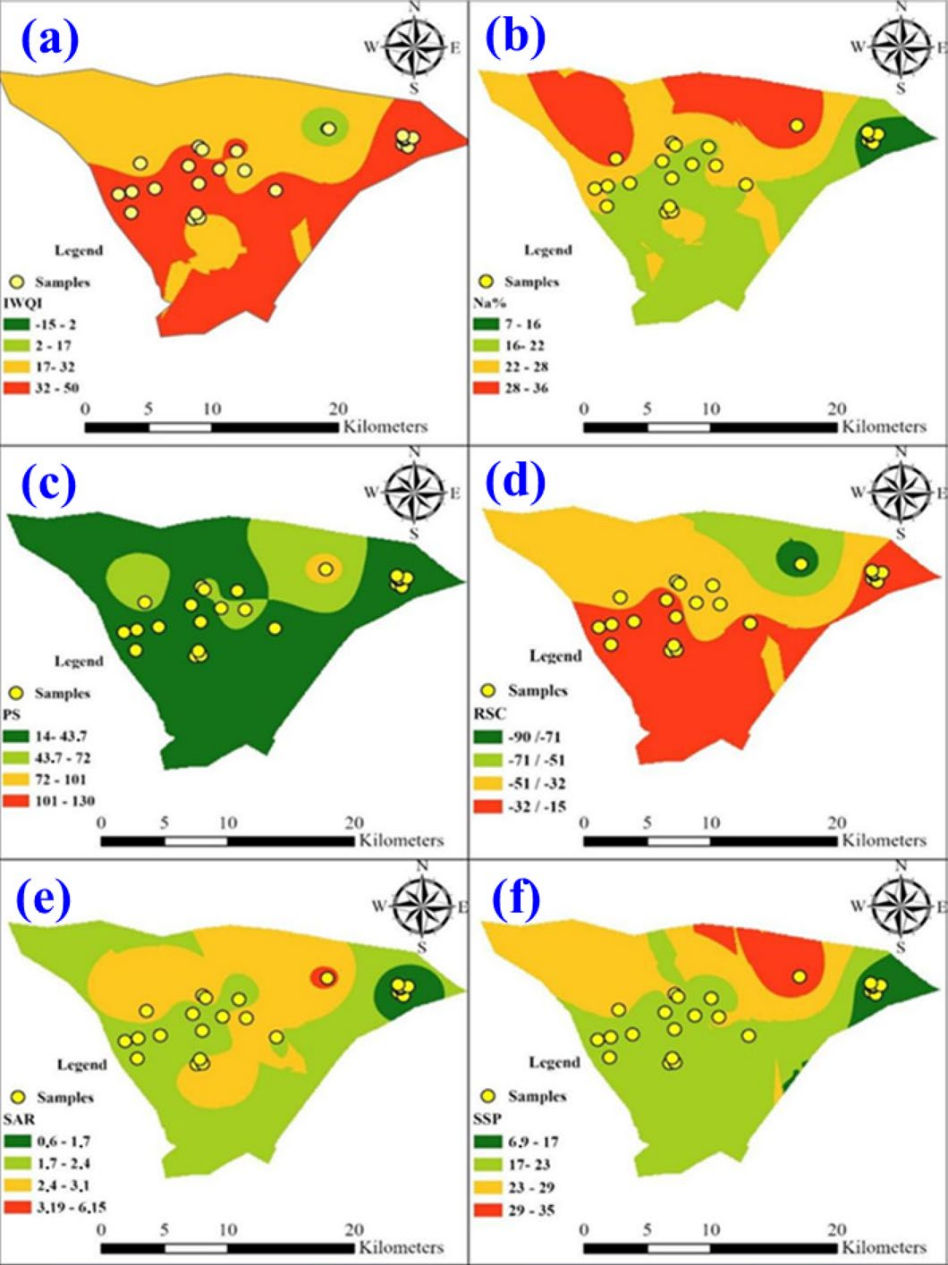
Spatial Distribution Map of **(a)** IWQI, **(b)** Na%, **(c)** PS, **(d)** RSC, **(e)** SAR, and **(f)** SSP after using EBKRP method. Map created using ArcGIS Pro 2.8.8 (Esri; https://www.esri.com/arcgis/about-arcgis).

According to Fig. 15, a clear persistence of low-quality areas in the north is deduced, but with more precise boundaries and additional nuances in the quality gradient, notably unfavorable islands more distinctly identified in the center. This confirms the diagnosis of Fig. 14, while further highlighting the vulnerability of certain areas where the risk of salinization is high.

The EBKRP method allows for a much more gradual and localized decrease in SAR values across the region (Fig. 15e), which facilitates the identification of areas that are potentially affected by alkalization and reduced permeability issues due to excess sodium. The spatial distribution map of RSC provides more detailed information on the location of risks related to the presence of soluble carbonates, with maximum values in the northeast and center (Fig. 15d). The spatial distribution map of SSP defined areas of the highest proportions with soluble sodium in the northeast and center, which suggesting potential accumulation of soluble sodium in the soil, and posing risks to soil structural integrity (Fig. 15f). The comparison between Figs. 14 and 15 revealed that the EBKRP technique achieves a clear improvement through two mechanisms including refined spatial precision and optimized micro-risk zone identification. Thus, it allows more targeted management to prevent the risks of sodicity, reduced permeability, salinization, or alkalinization of soils, and therefore to adapt irrigation practices to the local reality of each sector. The superiority of the EBKRP method over the traditional IDW interpolation is quantitatively confirmed through cross-validation results (Table 7). The EBRK approach provides substantially lower error metrics (ME, RMSE, MAE) and a higher coefficient of determination (R²), reflecting better prediction reliability and accuracy.

**Table 7:** Cross-validation results of interpolation methods.

Method	ME	RMSE	MAE	*R*²
IDW	0.45	8.12	5.76	0.68
EBRK	0.05	4.23	3.15	0.87

According to a regression-kriging approach, the EBKRP method enhances interpolation accuracy by incorporating explanatory variable rasters^106^. Here, the spatial distribution maps derived from the EBKRP method (Fig. 15) demonstrated a significant heterogeneity in irrigation water quality across Ouled Djellal area. The IWQI indicated deterioration in water quality especially in the regions that affected by elevated levels of salinity and sodicity. Moreover, the high values of Na%, SAR, RSC, and SSP in several areas can be considered as a potential risk of soil degradation due to the accumulation of sodium, which will impact on soil structure and permeability. These findings emphasized the significance of specific water management strategies in Ouled Djellal in order to try to mitigate future hazards and to maintain viable irrigation water supplies over the long term.

#### IWQIs prediction using ANN, SVM and MLR

**Fig. 16:**
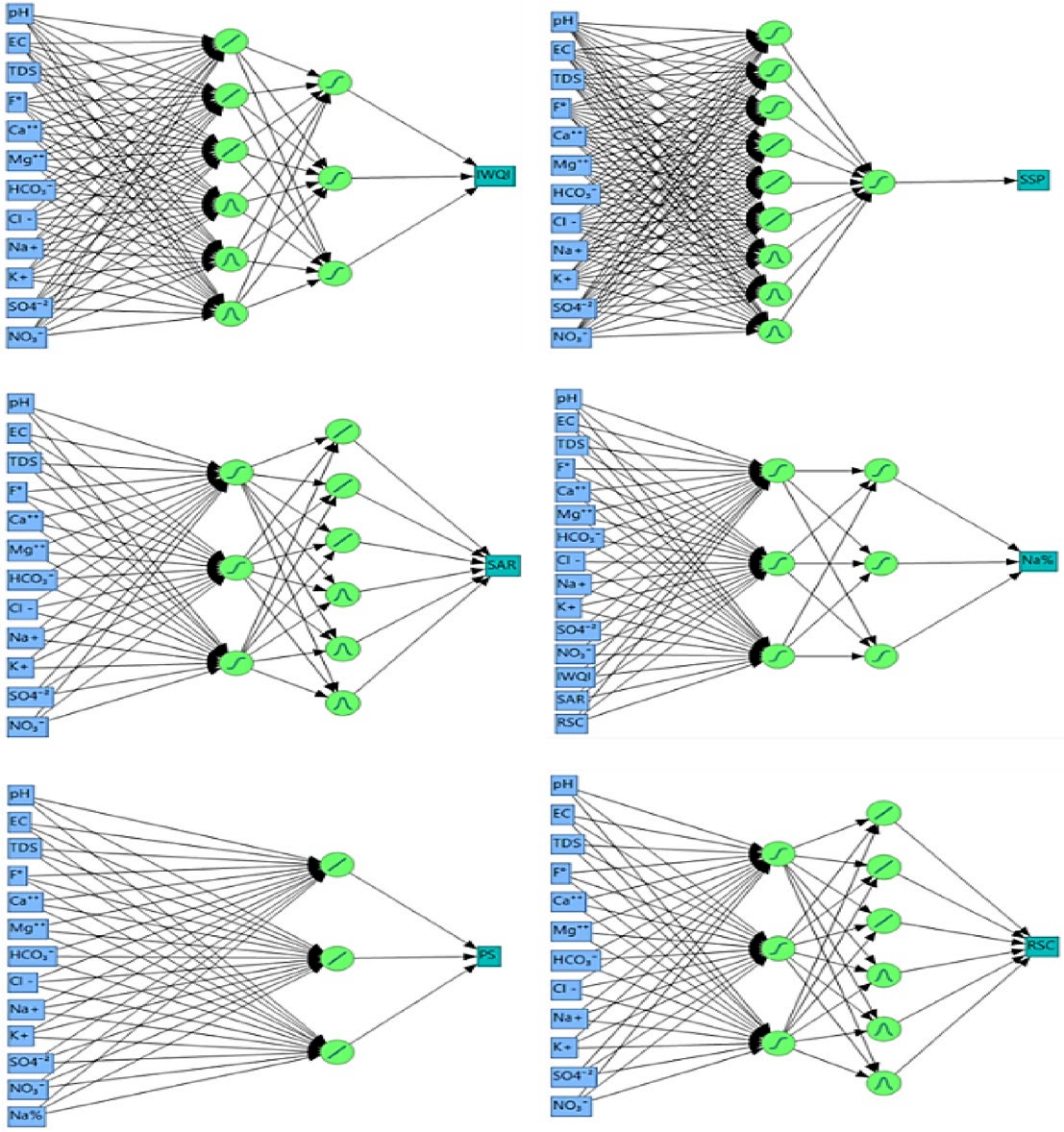
Number of Nodes for each type of activation in the ANN Model for the IWQIs indexes (SAR, IWQI, SSP, NA%, RSC, and PS).

**Fig. 17:**
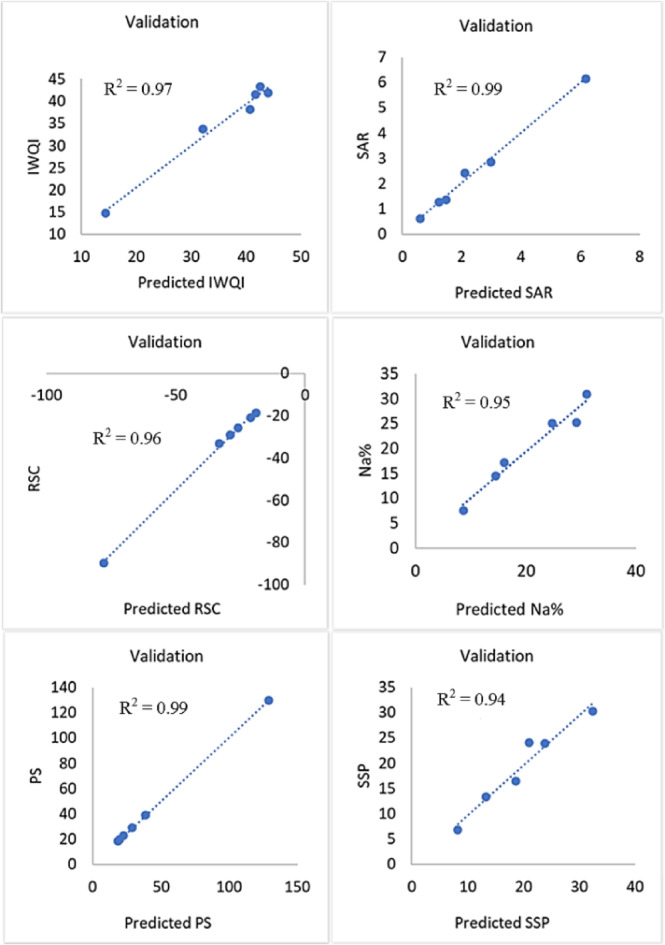
Application of the ANN model to validate output dataset for 6 indexes.

In this study, a comparative evaluation of ANN, SVM, and MLR models to predict water quality indices was realized; it revealed distinct limitations and strengths rooted in the capacity of each method to capture linear and nonlinear relationships. A number of artificial neural network (ANN) topologies designed to predict different water quality indices utilizing physicochemical factors as inputs are shown in the Fig. 16. A unique ANN model designed for a particular output index, such as the IWQI, SSP, SAR, Na, PI, and RSC, is represented by each subfigure. Measured water quality parameters including pH, EC, TDS, F⁻, Ca²⁺, Mg²⁺, HCO_3_⁻, Cl⁻, Na⁺, K⁺, SO_4_²⁻, and NO_3_⁻ make up the input layer, which is displayed on the left with blue boxes. Green neurons, which represent one or more hidden layers and capture nonlinear interactions between the variables, process these inputs. The validation outcomes of the ANN models created to forecast several IWQIs are shown in Fig. 17. With R^2^ values ranging from 0.94 to 0.99, all models show substantial correlations between anticipated and observed values, indicating exceptional predictive accuracy. In addition to Na% (R² = 0.95) and SSP (R² = 0.94), the WQI (R² = 0.97), SAR (R² = 0.99), PS (R² = 0.99), and RSC (R² = 0.96) models exhibit exceptionally good accuracy.

**Fig. 18:**
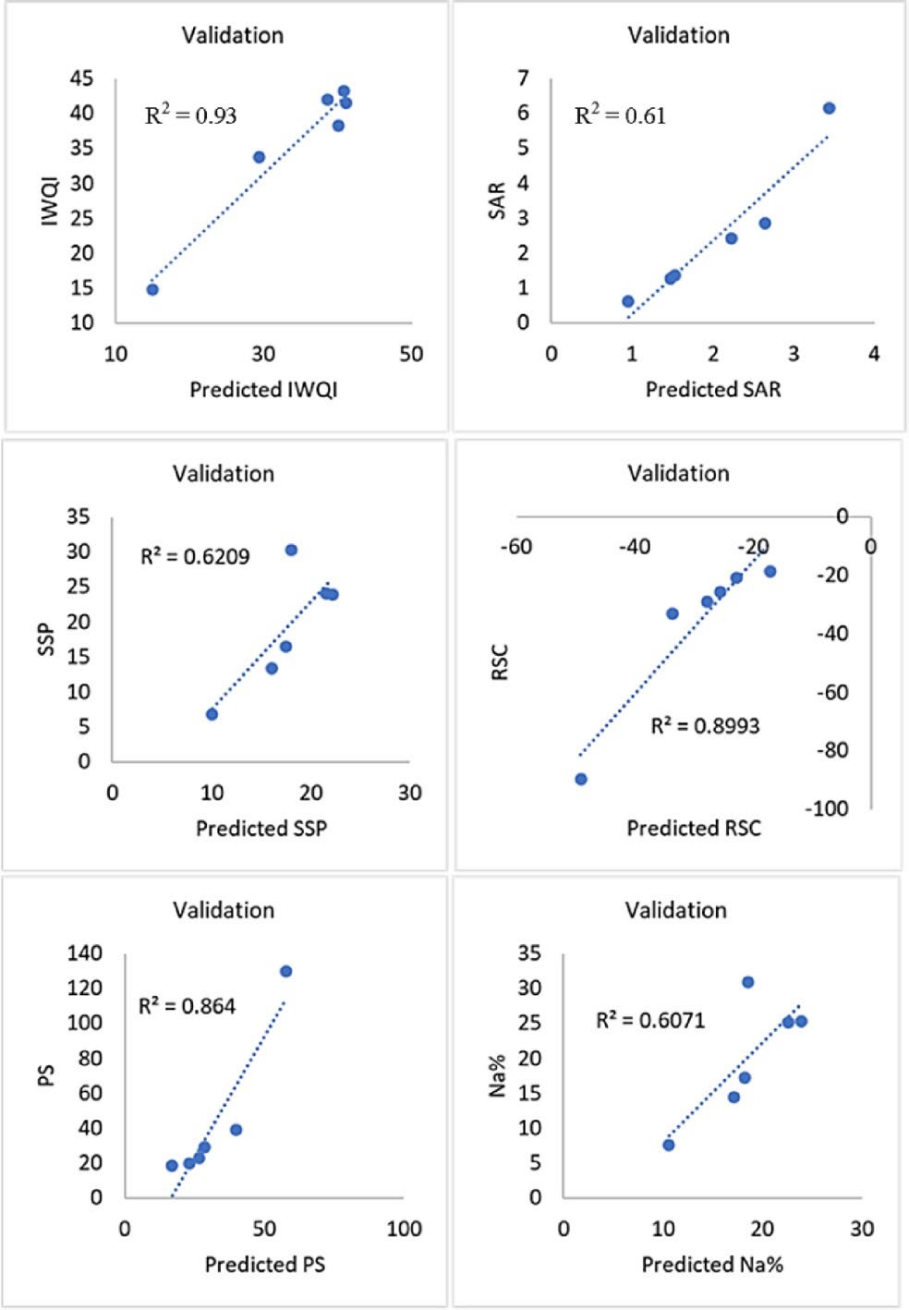
Application of the SVM model to validate output dataset for 6 indexes.

**Fig. 19:**
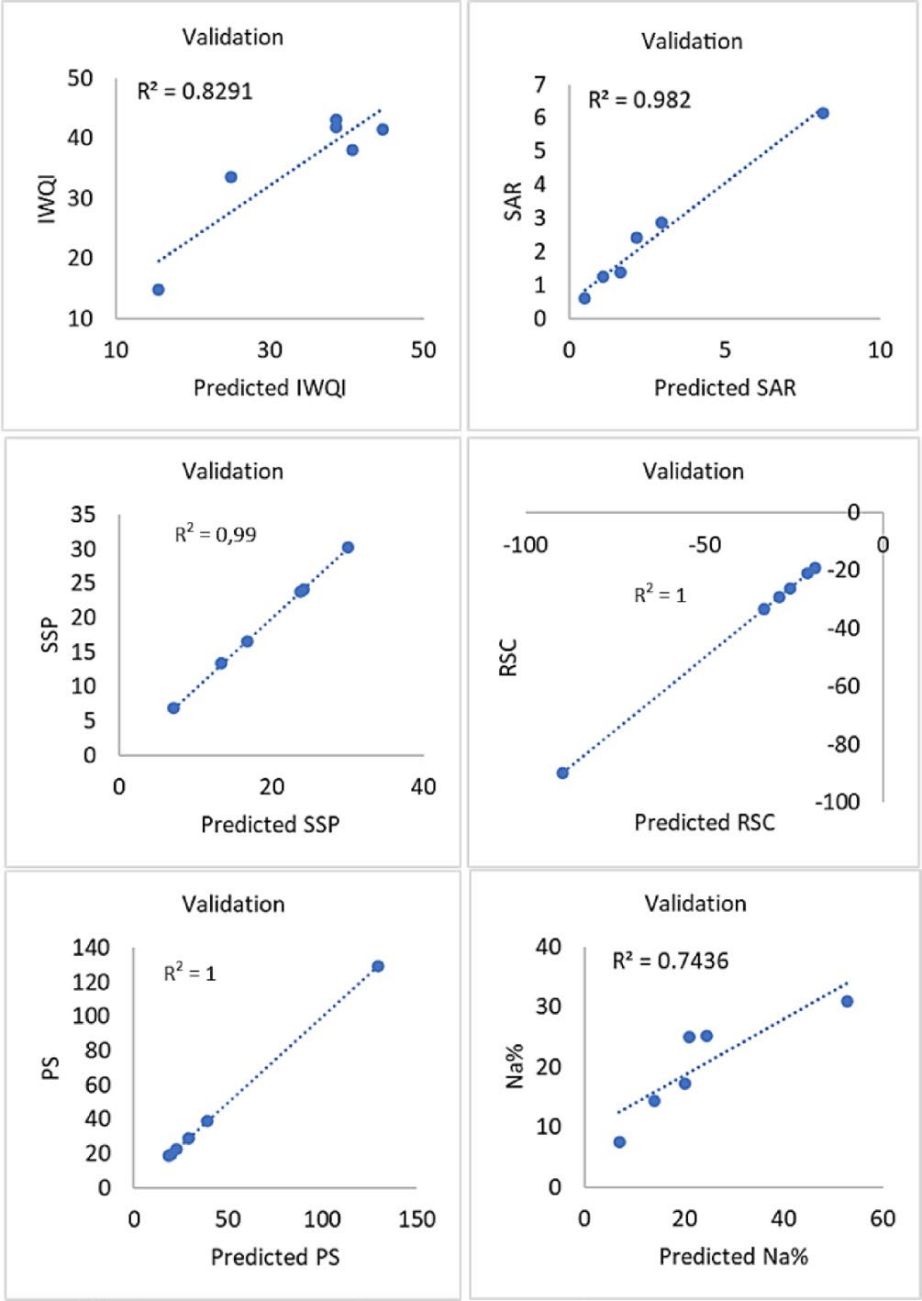
Application of the MLR model to validate output dataset for sex indexes.

In addition, strong correlations between IWQI (R² = 0.93) and RSC (R² = 0.90) among the SVM models indicate dependable model performance (Fig. 18). While the models for SAR (R² = 0.61), SSP (R² = 0.62), and Na% (R² = 0.61) show rather poor predictive ability, PS (R² = 0.86) likewise shows respectable accuracy. Overall, even though the SVM models were able to predict the majority of indices with respectable accuracy, bigger training datasets and additional tuning could be needed to enhance the prediction performance for indices like SAR, SSP, and Na%. On the other hand, the findings of the MLR models demonstrate exceptional accuracy (R² > 0.98) for forecasting SAR, SSP, PS, RSC, and SAP, indicating that the developed model is very dependable for these parameters (Fig. 19). It exhibits moderate-to-excellent performance for sodium percentage (Na%, R² = 0.74) and good performance for the general water quality index (R² = 0.83). For further information, each model’s performance was evaluated using root mean square error (RMSE) and coefficient of determination on both training and validation datasets for IWQIs, which are compiled in Table 8.

As summarized in Table 8, the MLR approach scored almost perfect results (R² = 1.00; RMSE ≈ 0) for linearly governed indices, such as PS and RSC in the training phase, underscoring strong linear associations. Nevertheless, its predictive generalization proved limited: validation R² values declined for IWQI (R² = 0.82, RMSE = 4.56) and Na% (R² = 0.74, RMSE = 9.16), indicating an inability to accommodate the intrinsic nonlinearities inherent in certain hydrochemical interactions. For instance, in the study of^106^ in Sidi El Hani aquifer (central-eastern Tunisia), where MLR model was satisfactory but less accurate than an ANN model, Whereas R^2^, RMSE, and Mean Absolute Error (MAE) were 0.81, 1.20, and 1.63 for MLR Vs. 0.92, 1.02 and 0.90 for ANN. In general, MLR provided a low performed compared to other ML techniques^31,107^ in hydrological and environmental modeling tasks.

Concerning the SVM model, it demonstrated an optimal balance between bias and variance. Validation (R²) values were uniformly high across indices, such as IWQI (R^2^= 0.93, RMSE = 2.56), SAR (R^2^= 0.98), PS (R^2^= 0.99), and SSP (R^2^= 0.99), which reflecting robust model generalization. However, a high value of RMSE for RSC (16.56) suggests occasional sensitivity to outliers or complex non-monotonic behavior, warranting further kernel or hyperparameter optimization to fully harness SVM nonlinear mapping capabilities. For instance^67^, demonstrated that Weight of Evidence (WOE) exhibited a higher groundwater potential than SVM model according to their study in Pakistan, while^108^ showed that XGBoost obtained a better accuracy than SVM, even if both studies stated that SVM is still a good model for water quality assessment. Conversely, our results align with the findings of^107^ which showed that SVM model was superior than MLR and Propagation Neural Network (BPNN) for predicting of dissolved oxygen.

In our case, the ANN model outperformed in most validation scenarios, which achieving R² = 0.97 (RMSE = 1.50) for IWQI, and R² ≥ 0.99 for SAR and PS, thereby demonstrating exceptional aptitude for modeling intricate multivariate dependencies. Near-perfect training scores across all indices (R² ≈ 1.00; RMSE ≈ 0) attest to the network’s flexibility, though slight overfitting, which demonstrated by an increased validation for RSC (RMSE = 4.81), emphasizing the necessity of rigorous regularization or a higher data volume in future implementations. According to previous studies^109^, the model performance of the ANN model outperformed like SVM, which used for quality prediction of river water in Malaysia. Nevertheless, another studies reported a contrasting result. For example^110^, reported that SVM was the most accurate model compared to ANN to predict water quality in Tireh river, IRAN. Similarly^111^, indicated that the prediction result of SVM was superior to ANN model in precision and generalization ability for groundwater depth prediction in Mengcheng Country, China. Finally, the limitations of this study represented in a larger dataset may improve the resilience and precision of the machine learning predictions, even while the sample size is sufficient for the models being employed. Furthermore, the study focusses on physicochemical aspects rather than analyzing specific pollutants in depth, such as dangerous heavy metals, which might provide a more complete picture of anthropogenic stress. This study emphasizes the need for further work such as expanding the geographical area, increasing the number of samples, conducting multi-seasonal sampling campaigns, developing dynamic modelling approaches such as integrating Internet of Things (IoT) for real-time monitoring and conservation strategies.

**Table 8:** The outcomes of ANN, SVM and MLR models.

Index	Model	Training		Validation	
		RMSE	R^2^	RMSE	R^2^
IWQI	ANN	3.54	0.85	1.50	0.97
	SVM MLR	0.91 1.69	0.99 0.96	2.56 4.56	0.93 0.82
SAR	ANN	1.29E-07	1	0.14	0.99
	SVM MLR	0.12 0.03	0.99 0.99	1.13 0.60	0.61 0.98
Na%	ANN	0.23	0.99	1.71	0.95
	SVM MLR	0.78 0.65	0.99 0.99	5.47 9.16	0.60 0.74
SSP	ANN	1.05	0.98	1.79	0.94
	SVM MLR	0.77 0.08	0.99 0.99	5.47 0.210	0.62 0.99
PS	ANN	1.70E-03	1	1.23E-03	0.99
	SVM MLR	0.77 0.08	0.99 0.99	5.47 0.21	0.62 0.99
RSC	ANN	0.003	0.99	4.81	0.96
	SVM MLR	1.08 4.34E-13	0.99 1	16.56 5.57E-12	0.89 1

## Conclusion

This study aimed to assess the groundwater (GW) quality of the phreatic aquifer using WQIs supported with ML models in the Ouled Djellal region, Algeria, It focused on identifying the principal geochemical mechanisms that controlled the acquisition of the GW chemistry and assessing their suitability for irrigation. This work combined chemical analyses of major ions, such as Ca²⁺, Mg²⁺, Na⁺, K⁺, SO4²⁻, HCO_3_⁻, Cl⁻, and NO_3_⁻ which conducted on 23 collected samples from representative boreholes. Hydrochemical characterization was complemented by chloro-alkaline indices (CAI), irrigation water quality indices (IWQs), such as IWQI, SAR, Na%, SSP, PS, RSC. Moreover, advanced multivariate statistics, such as principal component analysis and hierarchical cluster analysis were applied to discriminate sources of variability, classify water types. The results revealed dominant hydrochemical facies including calcium and magnesium chloride due to the influence of salinization and ion exchanges in the aquifer. The results of physicochemical analysis showed that the majority of the collected GW samples exhibited high mineralization, that ranging from 2566 to 16,100 µS/cm, with an average of 6994 µS/cm, due to geogenic processes (water-rock interaction and high evaporation) and the impact of human activities (intensive use of agricultural inputs). The results of IWQI revealed that about more than 60% of the samples had a restricted average quality for irrigation. The ML models, particularly the Artificial Neural Network (ANN) had the better accuracy, which showed a determination coefficient R² = 0.97 and RMSE = 1.50 in forecasting the IWQI, as well as R² values equal to or greater than 0.99 for SAR and PS, that demonstrating the robustness of the approach. Finally, integrating water quality indices and machine learning models supported with GIS techniques provides a robust, repeatable framework for effective groundwater quality forecasting, and provide a clear image for managing water quality and appropriate agriculture.

## Data Availability

Data presented or analyzed in this study are available from the corresponding author upon reasonable request.

## References

[1] Abdelkarim, B., Antunes, I. M. H. R., Abaab, N. & Agoubi, B. Modeling groundwater recharge mechanisms in semi-arid regions: integration of hydrochemical and isotopic data. *Euro-Mediterr J. Environ. Integr.* **8**, 893–905 (2023).

[2] Liu, N., Chen, M., Gao, D., Wu, Y. & Wang, X. Identification of hydrogeochemical processes in shallow groundwater using multivariate statistical analysis and inverse geochemical modeling. *Environ. Monit. Assess.* **197**, 135 (2025). 39760766 10.1007/s10661-024-13528-8

[3] Gad, M. et al. Comprehensive evaluation and prediction of groundwater quality and risk indices using quantitative approaches, multivariate analysis, and machine learning models: an exploratory study. *Heliyon* **10**, e36606 (2024). 10.1016/j.heliyon.2024.e36606 PMC11388788 39263076

[4] Touati, B. et al. Integrated analysis of precipitation and runoff trends in the Wadi Bouhamdane Basin, NE Algeria. *Mediterr. Geosci. Rev.* **7**, 159–179 (2025).

[5] Ghodbane, M. et al. Analysis of groundwater quality in the lower soummam Valley, North-East of Algeria. *J. Water Land. Dev.* **54**, 1–12 (2022). https://journals.pan.pl/dlibra/publication/141549/edition/124427 (2022).

[6] Karandish, F., Liu, S. & de Graaf, I. Global groundwater sustainability: A critical review of strategies and future pathways. *J. Hydrol.* **657**, 133060 (2025).

[7] Bouchaou, L., El Alfy, M., Shanafield, M., Siffeddine, A. & Sharp, J. Groundwater in arid and Semi-Arid areas. *Geosciences* **14**, 332 (2024).

[8] Reghais, A. et al. Risk assessment of potentially toxic elements and mapping of groundwater pollution indices using soft computer models in an agricultural area, Northeast Algeria. *J. Hazard. Mater.* **491**, 137991 (2025). 40147128 10.1016/j.jhazmat.2025.137991

[9] Alao, J. O., Bello, A., Lawal, H. & Abdullahi, D. Assessment of groundwater challenge and the sustainable management strategies. *Results Earth Sci.* **2**, 100049 (2024).

[10] Bordbar, M., Busico, G., Sirna, M., Tedesco, D. & Mastrocicco, M. A multi-step approach to evaluate the sustainable use of groundwater resources for human consumption and agriculture. *J. Environ. Manage.* **347**, 119041 (2023). 37783086 10.1016/j.jenvman.2023.119041

[11] ATHAMENA, A. et al. Origin, evolution and assessment of the hydrogeochemical functioning of a thermal mineral spring in Batna (Eastern Algeria). *Sci. Notes Sumy State Pedagog Univ. Named Makarenko Geogr. Sci.* **2**, 3–18 (2025).

[12] Belalite, H., Menani, M. R. & Athamena, A. Calculation of water needs of the main crops and water resources available in a semi-arid climate, case of Zana-Gadaïne plain, Northeastern Algeria. *Algerian J. Environ. Sci. Technol* **8**, 2 (2022).

[13] Reghais, A., Drouiche, A., Zahi, F. & Debieche, T. H. Hydrogeochemical evaluation of the terminal complex aquifer system in an arid area: a case study from the Biskra region, north-east Algeria. *Environ. Earth Sci.* **82**, 182 (2023).

[14] Mukherjee, A., Jha, M. K., Kim, K. W. & Pacheco, F. A. L. Groundwater resources: challenges and future opportunities. *Sci. Rep.* **14**, 28540 (2024). 39557953 10.1038/s41598-024-79936-5 PMC11574173

[15] Reghais, A., Drouiche, A., Ugochukwu, E., Zahi, F. & Debieche, T. H. Compositional data analysis (CoDA) and geochemical signatures of the terminal complex aquifer in an arid zone (northeastern Algeria). *J. Afr. Earth Sci.* **210**, 105162 (2024).

[16] Eid, M. H. et al. Impacts and sources of potential toxic elements on water quality and optimizing machine learning models for sustainable management. *Model. Earth Syst. Environ.* **11**, 375 (2025).

[17] Huang, Y. et al. Agricultural irrigation water price apportionment and sharing. *Water Policy*. **25**, 429–445 (2023).

[18] Gao, S., Zheng, T., Zheng, X. & Walther, M. Influence of layered heterogeneity on nitrate enrichment induced by cut-off walls in coastal aquifers. *J. Hydrol.* **609**, 127722 (2022).

[19] Hfaiedh, E. et al. Hydrogeochemical characterization and water quality evaluation associated with toxic elements using indexing approaches, multivariate analysis, and artificial neural networks in Morang, Tunisia. *Environ. Earth Sci.* **84**, 361 (2025).

[20] Bouselsal, B. & Saibi, H. Evaluation of groundwater quality and hydrochemical characteristics in the shallow aquifer of El-Oued region (Algerian Sahara). *Groundw. Sustain. Dev.* **17**, 100747 (2022).

[21] Al-Falal, A. N. A. et al. Aquatic system assessment of potentially toxic elements in El Manzala Lake, egypt: A statistical and machine learning approach. *Results Eng.* **26**, 105027 (2025).

[22] Gad, M. et al. New approach to predict wastewater quality for irrigation utilizing integrated indexical approaches and hyperspectral reflectance measurements supported with multivariate analysis. *Sci. Rep.* **15**, 16395 (2025). 40355568 10.1038/s41598-025-01181-1 PMC12069722

[23] Ehteram, M., Ahmed, A. N., Sherif, M. & El-Shafie, A. An advanced deep learning model for predicting water quality index. *Ecol. Indic.* **160**, 111806 (2024).

[24] Palabıyık, S. & Akkan, T. Evaluation of water quality based on artificial intelligence: performance of multilayer perceptron neural networks and multiple linear regression versus water quality indexes. *Environ. Dev. Sustain.*10.1007/s10668-024-05075-6 (2024).

[25] Bordbar, M., Busico, G., Stevenazzi, S. & Mastrocicco, M. How do hydrogeological and socio-economic parameters influence the likelihood of NO3 – pollution and Cl– salinization? An application within the campania region (Italy). *Nat. Hazards*. **121**, 12887–12907 (2025).

[26] Shams, M. Y. et al. Water quality prediction using machine learning models based on grid search method. *Multimed Tools Appl.* **83**, 35307–35334 (2024).

[27] Hassan, M. M. et al. Efficient prediction of water quality index (WQI) using machine learning algorithms. *Hum. -Centric Intell. Syst.* **1**, 86–97 (2021).

[28] Kouadri, S., Elbeltagi, A., Islam, A. R., Md., T. & Kateb, S. Performance of machine learning methods in predicting water quality index based on irregular data set: application on illizi region (Algerian southeast). *Appl. Water Sci.* **11**, 190 (2021).

[29] Trabelsi, F., Bel, H. & Ali, S. Exploring machine learning models in predicting irrigation groundwater quality indices for effective decision making in Medjerda river Basin, Tunisia. *Sustainability* **14**, 2341 (2022).

[30] Khodja, H. D., Aichour, A., Metaiche, M. & Ferhati, A. Hydrochemical assessment of Albian waters ofsouthern Algeria using water quality indices. *Pol. J. Environ. Stud.* **34**, 5599–5605 (2025).

[31] M’nassri, S., El Amri, A., Nasri, N. & Majdoub, R. Estimation of irrigation water quality index in a semi-arid environment using data-driven approach. *Water Supply*. **22**, 5161–5175 (2022).

[32] Jafar, R. et al. Multiple linear regression and machine learning for predicting the drinking water quality index in Al-Seine lake. *Smart Cities*. **6**, 2807–2827 (2023).

[33] Gaagai, A. et al. Application of water quality Indices, machine learning Approaches, and GIS to identify groundwater quality for irrigation purposes: A case study of Sahara Aquifer, Doucen Plain, Algeria. *Water* **15**, 289 (2023).

[34] Cornet, A. Introduction à l’hydrogéologie saharienne; par Cornet, André:: Gut | books4less (Versandantiquariat Petra Gros GmbH & Co. KG). (1964). https://www.abebooks.fr/Introduction-lhydrog%C3%A9ologie-saharienne-Cornet-Andr%C3%A9/20665435176/bd (1964).

[35] Ahcène, S., Bachir, H. & Bourafai, S. Hydrochemical characteristics of aquifers and their predicted impact on soil properties in Biskra region, Algeria. *Egypt. J. Agric. Res.*10.21608/ejar.2021.56750.1068 (2021).

[36] Athamena, A. et al. Chemometrics of the environment: hydrochemical characterization of groundwater in Lioua plain (North Africa) using time series and multivariate statistical analysis. *Sustainability* **15**, 20 (2023).

[37] Guettaia, S., Hacini, M., Boudjema, A. & Zahrouna, A. Vulnerability assessment of an aquifer in an arid environment and comparison of the applied methods: case of the mio-plio-quaternary aquifer. *Energy Procedia*. **119**, 482–489 (2017).

[38] Sadooni, F. N. & Alsharhan, A. S. Regional stratigraphy, facies distribution, and hydrocarbons potential of the oligocene strata across the Arabian plate and Western Iran. *Carbonates Evaporites*. **34**, 1757–1770 (2019).

[39] Rodier, J. *L’analyse De L’eau: Eaux Naturelles, Eaux Résiduaires, Eau De Mer: Chimie, Physico-Chimie, Bactériologie, Biologie* (Dunod, 1984).

[40] Lima, V. R. et al. Nopalea cochenillifera Biomass as Bioadsorbent in Water Purification. *Water* 13, (2021). (2012).

[41] Szekely, E. A rapid colorimetric method for analysis of nitrate nitrogen by reduction to nitrite. *Commun. Soil. Sci. Plant. Anal.* **22**, 1295–1302 (1991).

[42] Rice, B. & Eaton *Standard Methods for the Examination of Water and Wastewater, 23rd Edition: Rice, E.W., Baird, R.B., Eaton, A.D.: 9780875532875*:American Public Health Association, (2017).

[43] Cho, Y. C., Choi, H., Lee, M. G., Kim, S. H. & Im, J. K. Identification and apportionment of potential pollution sources using multivariate statistical techniques and APCS-MLR model to assess surface water quality in Imjin river watershed, South Korea. *Water* **14**, 793 (2022).

[44] Al-Mashreki, M. H. et al. Integration of geochemical Modeling, multivariate Analysis, and irrigation indices for assessing groundwater quality in the Al-Jawf Basin, Yemen. *Water* **15**, 1496 (2023).

[45] Ibrahim, H. et al. Evaluation and prediction of groundwater quality for irrigation using an integrated water quality Indices, machine learning models and GIS approaches: A representative case study. *Water* **15**, 694 (2023).

[46] Chounlamany, V., Tanchuling, M. A. & Inoue, T. Spatial and Temporal variation of water quality of a segment of Marikina river using multivariate statistical methods. *Water Sci. Technol.* **76**, 1510–1522 (2017). 28953477 10.2166/wst.2017.279

[47] Mohanty, C. R. & Nayak, S. K. Assessment of seasonal variations in water quality of Brahmani river using PCA. *Adv. Environ. Res.* **6**, 53–65 (2017).

[48] Wu, M. L. & Wang, Y. S. Using chemometrics to evaluate anthropogenic effects in Daya Bay, China. *Estuar. Coast Shelf Sci.* **72**, 732–742 (2007).

[49] Schoeller, H. Methods and techniques of groundwater investigation and development. *Water Resour. Ser* **33**, 44–52 (1967).

[50] Meireles, A. C. M., de Andrade, E. M., Chaves, L. C. G. & Frischkorn, H. Crisostomo, L. A. A new proposal of the classification of irrigation water. *Rev. Ciênc Agronômica*. **41**, 349–357 (2010).

[51] Richards, L. A. *Diagnosis and Improvement of Saline and Alkali Soils* Vol. 78 (LWW, 1954).

[52] Ravikumar, P., Aneesul Mehmood, M. & Somashekar, R. K. Water quality index to determine the surface water quality of Sankey tank and Mallathahalli lake, Bangalore urban district, Karnataka, India. *Appl. Water Sci.* **3**, 247–261 (2013).

[53] Eaton, F. M. Significance of carbonates in irrigation waters. *Soil. Sci.* **69**, 123–134 (1950).

[54] Doneen, L. D. Water quality for agriculture, department of irrigation. *Univ Calif. Davis* **48**, 4 (1964).

[55] Rammohan, B., Partheeban, P., Ranganathan, R. & Balaraman, S. Groundwater Quality Prediction and Analysis Using Machine Learning Models and Geospatial Technology. *Sustainability* 16, (2024).

[56] Ismail, R. et al. Artificial intelligence for application in water engineering: the use of ANN to determine water quality index in rivers. *Civ. Eng. J.* **10**, 2261–2274 (2024).

[57] Chen, Y. et al. Artificial neural networks in the prediction and assessment for water quality: A review. *J. Phys. Conf. Ser.* **1237**, 042051 (2019).

[58] Masoud, M., El Osta, M., Alqarawy, A., Elsayed, S. & Gad, M. Evaluation of groundwater quality for agricultural under different conditions using water quality indices, partial least squares regression models, and GIS approaches. *Appl. Water Sci.* **12**, 244 (2022).

[59] Gad, M. et al. Groundwater quality and health risk assessment using indexing Approaches, multivariate statistical Analysis, artificial neural Networks, and GIS techniques in El Kharga Oasis. *Egypt. Water*. **15**, 1216 (2023).

[60] Kushwaha, N. L. Stacked hybridization to enhance the performance of artificial neural networks (ANN) for prediction of water quality index in the Bagh river basin, India. *Heliyon,* **10**, e31085 (2024). 10.1016/j.heliyon.2024.e31085 PMC11112320 38784559

[61] Chen, Y., Song, L., Liu, Y., Yang, L. & Li, D. A review of the artificial neural network models for water quality prediction. *Appl. Sci.* **10**, 5776 (2020).

[62] Banda, T. D. & Kumarasamy, M. Artificial neural network (ANN)-Based water quality index (WQI) for assessing Spatiotemporal trends in surface water quality—A case study of South African river basins. *Water* **16**, 1485 (2024).

[63] Ansari, A. T., Nigar, N., Faisal, H. M. & Shahzad, M. K. AI for clean water: efficient water quality prediction leveraging machine learning. *Water Pract. Technol.* **19**, 1986–1996 (2024).

[64] Arefinia, A. et al. Using support vector machine (SVM) in modeling water resources systems. In *Computational Intelligence for Water and Environmental Sciences* (eds Bozorg-Haddad, O., Zolghadr-Asli, B. et al.) 179–199 (Springer Nature, 2022). 10.1007/978-981-19-2519-1_9.

[65] Singh, K. P., Basant, N. & Gupta, S. Support vector machines in water quality management. *Anal. Chim. Acta*. **703**, 152–162 (2011). 21889629 10.1016/j.aca.2011.07.027

[66] Yahya, A. S. et al. Water quality prediction model based support vector machine model for ungauged river catchment under dual scenarios. *Water* **11**, 1231 (2019).

[67] Riaz, M. T. et al. An integrated approach of support vector machine (SVM) and weight of evidence (WOE) techniques to map groundwater potential and assess water quality. *Sci. Rep.* **14**, 26186 (2024). 39478094 10.1038/s41598-024-76607-3 PMC11525780

[68] Dimple, D., Rajput, J., Al-Ansari, N. & Elbeltagi, A. Predicting Irrigation Water Quality Indices Based on Data-Driven Algorithms: Case Study in Semiarid Environment. *J. Chem.* **2022**, 4488446 (2022).

[69] Akhlaq, M. et al. Comparative analysis of machine learning algorithms for water quality prediction. *Tellus Dyn. Meteorol. Oceanogr* **76**, 177–192 (2024).

[70] Gai, R. & Guo, Z. A water quality assessment method based on an improved grey relational analysis and particle swarm optimization multi-classification support vector machine. Front. Plant Sci. 14 (2023). 10.3389/fpls.2023.1099668. 10.3389/fpls.2023.1099668 PMC9905719 36760628

[71] Brix, K. V., DeForest, D. K., Tear, L., Grosell, M. & Adams, W. J. Use of multiple linear regression models for setting water quality criteria for copper: A complementary approach to the biotic ligand model. *Environ. Sci. Technol.* **51**, 5182–5192 (2017). 28409924 10.1021/acs.est.6b05533

[72] Fernandes, P., Fonseca, A. C. R. & Pacheco, A. Sanches Fernandes, L. F. Water quality predictions through linear regression - A brute force algorithm approach. *MethodsX* **10**, 102153 (2023). 37077896 10.1016/j.mex.2023.102153 PMC10106967

[73] Su, K. et al. Water quality evaluation based on water quality index and multiple linear regression: A research on Hanyuan lake in Southern Sichuan Province, China. *Water Environ. Res.* **96**, e11055 (2024). 38804065 10.1002/wer.11055

[74] Zare Abyaneh, H. Evaluation of multivariate linear regression and artificial neural networks in prediction of water quality parameters. *J. Environ. Health Sci. Eng.* **12**, 40 (2014). 24456676 10.1186/2052-336X-12-40 PMC3906747

[75] Malone, B. P., Styc, Q., Minasny, B. & McBratney, A. B. Digital soil mapping of soil carbon at the farm scale: A Spatial downscaling approach in consideration of measured and uncertain data. *Geoderma* **290**, 91–99 (2017).

[76] Saggi, M. K. & Jain, S. Reference evapotranspiration Estimation and modeling of the Punjab Northern India using deep learning. *Comput. Electron. Agric.* **156**, 387–398 (2019).

[77] FAO. L’eau, l’agriculture et l’alimentation. (2004). https://www.fao.org/4/y4683f/y4683f00.htm

[78] Selvam, S., Venkatramanan, S., Chung, S. Y. & Singaraja, C. Identification of groundwater contamination sources in Dindugal district of Tamil Nadu, India using GIS and multivariate statistical analyses. *Arab. J. Geosci.* **9**, 407 (2016).

[79] Ghalib, H. B. Groundwater chemistry evaluation for drinking and irrigation utilities in East Wasit province, central Iraq. *Appl. Water Sci.* **7**, 3447–3467 (2017).

[80] Litvinovich, A., Pavlova, O., Lavrishchev, A., Bure, V. & Saljnikov, E. Magnesium leaching processes from sod-podzolic sandy loam reclaimed by increasing doses of finely ground dolomite. *Zemdirb -Agric*. **108**, 109–116 (2021).

[81] Ayers, R. S. & Westcot, D. W. Water quality for agriculture. (1994). https://www.fao.org/4/T0234e/T0234e00.htm

[82] Das, S. & Nag, S. K. Deciphering groundwater quality for irrigation and domestic purposes – a case study in Suri I and II blocks, birbhum District, West Bengal, India. *J. Earth Syst. Sci.* **124**, 965–992 (2015).

[83] Piper, A. M. A graphic procedure in the geochemical interpretation of water-analyses. *AGU J* (1944). https://agupubs.onlinelibrary.wiley.com/action/showCitFormats?doi=10.1029%2FTR025i006p00914

[84] Gibbs, R. J. Mechanisms controlling world water chemistry. *Science* **170**, 1088–1090 (1970). 17777828 10.1126/science.170.3962.1088

[85] Gaikwad, S. et al. Geochemical mobility of ions in groundwater from the tropical Western Coast of Maharashtra, india: implication to groundwater quality. *Environ. Dev. Sustain.* **22**, 2591–2624 (2020).

[86] Jain, C. K., Sharma, S. K. & Singh, S. Physico-chemical characteristics and hydrogeological mechanisms in groundwater with special reference to arsenic contamination in Barpeta District, Assam (India). *Environ. Monit. Assess.* **190**, 417 (2018). 29926193 10.1007/s10661-018-6781-5

[87] Xiao, J., Jin, Z. D., Wang, J. & Zhang, F. Hydrochemical characteristics, controlling factors and solute sources of groundwater within the Tarim river basin in the extreme arid region, NW Tibetan plateau. *Quat Int.* **380–381**, 237–246 (2015).

[88] Zhu, B. et al. Hydrogeochemistry of three watersheds (the Erlqis, Zhungarer and Yili) in Northern Xinjiang, NW China. *Appl. Geochem.* **26**, 1535–1548 (2011).

[89] Gaillardet, J., Dupré, B., Louvat, P. & Allègre, C. J. Global silicate weathering and CO2 consumption rates deduced from the chemistry of large rivers. *Chem. Geol.* **159**, 3–30 (1999).

[90] Apollaro, C. et al. Geochemical Modeling of Water-Rock Interaction Processes in the Pollino National Park. *Geofluids, ***2011,** 6655711 (2021).

[91] Athamena, A. & Menani, M. R. Nitrogen flux and hydrochemical characteristics of the calcareous aquifer of the Zana plain, North East of Algeria. *Arab. J. Geosci.* **11**, 356 (2018).

[92] Mayo, A. L. & Loucks, M. D. Solute and isotopic geochemistry and ground water flow in the central Wasatch Range, Utah. *J. Hydrol.* **172**, 31–59 (1995).

[93] Yousaf, M., Ali, O. M. & Rhoades, J. D. Dispersion of clay from some Salt-Affected, arid land soil aggregates. *Soil. Sci. Soc. Am. J.* **51**, 920–924 (1987).

[94] Masmoudi, A. Ӛ Théses-Algérie: Doctorat, Magister, Master… https://www.theses-algerie.com (2012).

[95] khezami, F. et al. Hydrogeochemical assessment and modeling of groundwater processes and pollution: a case study of the Grombalia aquifer in Northeast Tunisia. *Model. Earth Syst. Environ.* **10**, 3573–3592 (2024).

[96] Datta, P. S. & Tyagi, S. K. Major ion chemistry of groundwater in Delhi area: chemical weathering processes and groundwater flow regime. *J. Geol. Soc. India*. **47**, 179–188 (1996).

[97] Zhang, T. et al. Ion chemistry of groundwater and the possible controls within Lhasa river Basin, SW Tibetan plateau. *Arab. J. Geosci.* **11**, 510 (2018).

[98] Zahi, F., Drouiche, A., Medjani, F., Azzeddine, R. & Djidel, M. Hydrogeochemical processes controlling surface water quality for irrigation in a mediterranean wetland ecosystem, Northeast Algeria. *Environ. Monit. Assess.* **196**, 881 (2024). 39223287 10.1007/s10661-024-13019-w

[99] Li, P., Wu, J. & Qian, H. Preliminary assessment of hydraulic connectivity between river water and shallow groundwater and Estimation of their transfer rate during dry season in the Shidi River, China. *Environ. Earth Sci.* **75**, 99 (2016).

[100] ATHAMENA, A. Flux azoté: Origine et devenir dans les eaux souterraines cas de la région de Zana, Est algérien. (Université de Batna 2, (2018).

[101] Wu, J., Li, P. & Qian, H. Hydrochemical characterization of drinking groundwater with special reference to fluoride in an arid area of China and the control of aquifer leakage on its concentrations. *Environ. Earth Sci.* **73**, 8575–8588 (2015).

[102] Parkhurst, D. L. & Appelo, C. J. *User’s Guide to PHREEQC (Version 2): A Computer Program for Speciation, Batch-Reaction, One-Dimensional Transport, and Inverse Geochemical Calculations*. *Water-Resources Investigations Report* https://pubs.usgs.gov/publication/wri994259 (1999). 10.3133/wri994259

[103] Allen, H. N. The theory of electrolytes. *Sci. Prog Twent Century 1919–1933*. **18**, 471–473 (1924).

[104] Kraiem, Z., Zouari, K., Chkir, N. & Agoune, A. Geochemical characteristics of arid shallow aquifers in Chott Djerid, south-western Tunisia. *J. Hydro-Environ Res.* **8**, 460–473 (2014).

[105] Byrne, M. P. et al. Urease and nitrification Inhibitors—As mitigation tools for greenhouse gas emissions in sustainable dairy systems: A review. *Sustainability* **12**, 6018 (2020).

[106] Gribov, A. & Krivoruchko, K. Empirical bayesian kriging implementation and usage. *Sci. Total Environ.* **722**, 137290 (2020). 32208233 10.1016/j.scitotenv.2020.137290

[107] Li, X., Sha, J. & Wang, Z. A comparative study of multiple linear regression, artificial neural network and support vector machine for the prediction of dissolved oxygen. *Hydrol. Res.* **48**, 1214–1225 (2016).

[108] Yusri, H. I. H., Ab Rahim, A. A., Hassan, S. L. M. & Halim, I. S. A. & N. E. Abdullah. Water Quality Classification Using SVM And XGBoost Method. in *IEEE 13th Control and System Graduate Research Colloquium (ICSGRC)* 231–236 (2022). 231–236 (2022). (2022). 10.1109/ICSGRC55096.2022.9845143

[109] Najwa, M. et al. Comparison between regression Models, support vector machine (SVM), and artificial neural network (ANN) in river water quality prediction. *Processes* **10**, 1652 (2022).

[110] Haghiabi, A. H., Nasrolahi, A. H. & Parsaie, A. Water quality prediction using machine learning methods. *Water Qual. Res. J.* **53**, 3–13 (2018).

[111] Zhou, T., Wang, F. & Yang, Z. Comparative analysis of ANN and SVM models combined with wavelet preprocess for groundwater depth prediction. *Water* **9**, 781 (2017).

